# Data-driven prediction of microhardness and tensile strength in microwave-sintered ZrC reinforced AA7075/SiC hybrid composites using machine learning

**DOI:** 10.1038/s41598-026-46609-4

**Published:** 2026-04-03

**Authors:** E Srinath, K Venkateswara Reddy, Guttikonda Manohar, Pramod Kumar P, Nagaraj Ashok

**Affiliations:** 1 School of Computer Science and Artificial Intelligence, SR University, Warangal, 506371 Telangana India; 2 Department of Mechanical Engineering, CVR College of Engineering, Vastunagar, Hyderabad, 501510 Telangana India; 3 Faculty of Mechanical Engineering, Jimma Institute of Technology, Jimma University, Jimma, 378 Ethiopia

**Keywords:** Machine learning, Composite materials, Predictive modelling, Neural networks, Random Forest, Engineering, Materials science

## Abstract

The development of lightweight, high-strength materials is critical for next-generation aerospace and automotive applications. In this study, a comprehensive materials informatics framework is developed to predict the microhardness and tensile strength of microwave-sintered AA7075/SiC/ZrC hybrid composites. A structured experimental dataset comprising 172 samples was generated by systematically varying SiC and ZrC content, compaction pressure, sintering temperature, and sintering time, ensuring broad coverage of the processing space. Unlike conventional studies that rely solely on standalone machine learning implementations, the present work integrates advanced data visualization, rigorous model validation, and physically interpretable learning. Multiple regression algorithms—including Artificial Neural Networks (ANN), Extreme Gradient Boosting (XGBoost), Random Forest (RF), Support Vector Regression (SVR), and K-Nearest Neighbors (KNN)—were trained using optimized hyperparameters and evaluated through nested cross-validation to ensure robustness and generalizability. Among these, ANN and XGBoost demonstrated superior predictive performance, achieving coefficients of determination (R²) exceeding 0.97 for tensile strength and 0.95 for microhardness. A key novelty of this study lies in explicitly linking machine learning predictions with underlying metallurgical mechanisms. Feature importance analysis, supported by microstructural observations, reveals that tensile strength is predominantly governed by compaction pressure and reinforcement distribution, while microhardness is strongly influenced by SiC content and sintering parameters. These relationships are interpreted in terms of densification behavior, Orowan strengthening, and grain refinement mechanisms. By bridging experimental materials science with interpretable machine learning, this work provides a reliable and physically grounded predictive framework that reduces experimental effort and enables accelerated optimization of hybrid aluminium matrix composites.

## Introduction

The relentless quest for lighter, stronger and more damage-tolerant structural components in aerospace, automotive, defence and renewable-energy systems has propelled a decisive shift away from monolithic metals toward engineered composite materials^1,2^. By embedding high-modulus, thermally stable ceramics within ductile aluminium matrices, these composites achieve superior specific strength, stiffness and corrosion resistance while enabling weight reductions that translate directly into fuel savings and extended service life. Among aluminium grades, precipitation-hardenable AA7075 is a frequent matrix of choice because its Zn–Mg–Cu chemistry already delivers excellent baseline strength that can be further amplified by particulate reinforcement. Single-phase metal-matrix composites (MMCs), however, often confront trade-offs: additions that boost hardness may erode fracture toughness or wear resistance^3^. Hybrid MMCs circumvent these limitations by combining two or more ceramics whose complementary attributes act synergistically. Systems containing hard SiC for load bearing and wettable, thermally robust ZrC for interface stability exemplify this strategy; hybrids have demonstrated tensile-strength and hardness gains exceeding 20% relative to the monolithic alloy while maintaining excellent ductility^4,5^. The powder metallurgy route, particularly involving controlled compaction and sintering schedules, enables precise control over reinforcement distribution and interfacial bonding, delivering both performance and manufacturing consistency benefits^6^.

Yet each additional reinforcement weight fraction, compaction pressure, sintering temperature or sintering time multiplies the number of specimens needed for full mechanical characterisation. A modest design matrix of five SiC–ZrC weight fractions, four compaction pressures, three sintering temperatures and three hold times already demands one hundred eighty tensile and hardness tests, not counting replicates. Such factorial escalation consumes laboratory time, material and instrumentation budgets, delaying optimisation and industrial adoption. The resulting sparsity of well-curated experimental datasets has become a bottleneck for data-driven alloy design^7,8^.

Traditional statistical methods, such as linear and polynomial regression, are often limited in their ability to capture complex nonlinear interactions among multiple processing parameters. In contrast, machine learning approaches provide a flexible framework capable of modeling such nonlinear and coupled relationships without prior assumptions. In the present study, models such as ANN, Random Forest, and XGBoost are employed to effectively capture the intricate relationships between composition, processing conditions, and mechanical properties^9^. These models are particularly suitable for materials systems where multiple variables interact simultaneously, resulting in improved predictive accuracy and reliability^10,11^.

Artificial intelligence (AI) offers a pathway past this impasse. Recent reviews highlight how machine-learning (ML) algorithms, when coupled with domain knowledge and physics-based simulation, accelerate materials discovery, optimise process windows and underpin digital twins for adaptive manufacturing^12,13^. In metals research, ensemble learners, kernel methods and deep neural networks routinely capture non-linear, multivariate relationships that elude classical regression, opening possibilities for rapid, low-cost property prediction before any billet is cast or coupon machined^14,15^. Nevertheless, predictive modelling of heterogeneous, hybrid composites remain challenging. Reinforcements introduce multi-scale anisotropy, interfacial phases and residual porosity, all of which complicate constitutive behaviour. Sparse and noisy experimental data exacerbate overfitting, while transferability from laboratory coupons to full-scale components is seldom assessed. Reported successes—such as XGBoost regressors that achieved over 94% accuracy for powder metallurgy aluminium composites, neural networks that captured hardness and tensile strength trends in hybrid aluminium composites, and support-vector models that reproduced mechanical properties of sintered metal matrix composites within experimental scatter—confirm ML’s promise but also expose gaps in dataset size, feature engineering and interpretability^16,17^.

Recent literature demonstrates promising advances in ML-driven prediction of composite properties. Extreme gradient boosting (XGBoost) models have shown particular efficacy, with studies reporting R^2^ values exceeding 0.95 for predicting sintered density in Cu-Al alloys using compaction pressure, sintering temperature, and particle characteristics as inputs^18,19^. Similarly, artificial neural networks have successfully predicted tensile strength and hardness in hybrid aluminium matrix composites, achieving mean absolute percentage errors below 5%. Convolutional neural networks coupled with explainable AI techniques have demonstrated superior performance in capturing complex microstructure-property relationships in fiber-reinforced composites^20^. These advances underscore the potential of ML approaches to replace extensive experimental campaigns while providing interpretable insights into processing-property relationships^21,22^.

Recent advances in materials informatics have demonstrated the growing potential of machine learning techniques in predicting the mechanical behavior of composite materials^23^. Several studies have reported the successful application of machine learning algorithms in modeling the hardness and strength of polymer-based and nanocomposite systems, including polypropylene/carbon nanotube and polyethylene/carbon nanotube composites^24^. Similarly, predictive modeling of thermoplastic nanocomposites has shown that data-driven approaches can effectively capture complex process–property relationships^25,26^. Furthermore, emerging trends in sustainable composite development under Industry 4.0 and 5.0 frameworks emphasize the integration of data-driven methodologies for efficient material design and optimization^27^. These developments highlight the increasing importance of machine learning in modern composite research and provide a strong foundation for the present study.

Although several studies have employed machine learning for property prediction in composite materials, many remain limited to algorithmic performance without establishing a strong connection to underlying materials physics or ensuring model generalizability. In contrast, the present work introduces a structured materials informatics approach that integrates systematic experimental design, comprehensive data visualization, and rigorously validated machine learning models^28,29^. A key novelty of this study lies in bridging data-driven predictions with fundamental strengthening mechanisms—such as Orowan strengthening, grain refinement, and densification—supported by microstructural characterization^30,31^. Furthermore, the use of nested cross-validation and multi-model comparative analysis enhances the robustness and reliability of predictions. This combined experimental–computational framework provides not only high predictive accuracy but also physical interpretability, enabling more efficient and scientifically grounded optimization of AA7075/SiC/ZrC hybrid composites.

Therefore, the present study aims to (i) compile a comprehensive experimental database spanning reinforcement weight fractions of SiC and ZrC, compaction pressures, sintering temperatures and sintering times for AA7075 hybrid composites; (ii) benchmark multiple regression frameworks—including extreme-gradient boosting, random forests, support-vector regression and artificial neural networks—under nested cross-validation to ensure generalisability; (iii) elucidate the relative influence of reinforcement weight fraction, compaction pressure, sintering temperature and sintering time on hardness and tensile strength via advanced feature importance analysis; (iv) validate the best-performing models on unseen experimental data to compute confidence intervals; and (v) deliver an explainable, ML-driven predictive modelling framework for determining hardness and tensile strength of AA7075/SiC/ZrC hybrid composites. Achieving these objectives will demonstrate how AI can supplant labour-intensive trial-and-error campaigns, accelerating the deployment of lightweight, high-strength AA7075/SiC/ZrC hybrid composites in demanding service environments.

## Materials and methods

### Raw materials and fabrication

**Fig. 1:**
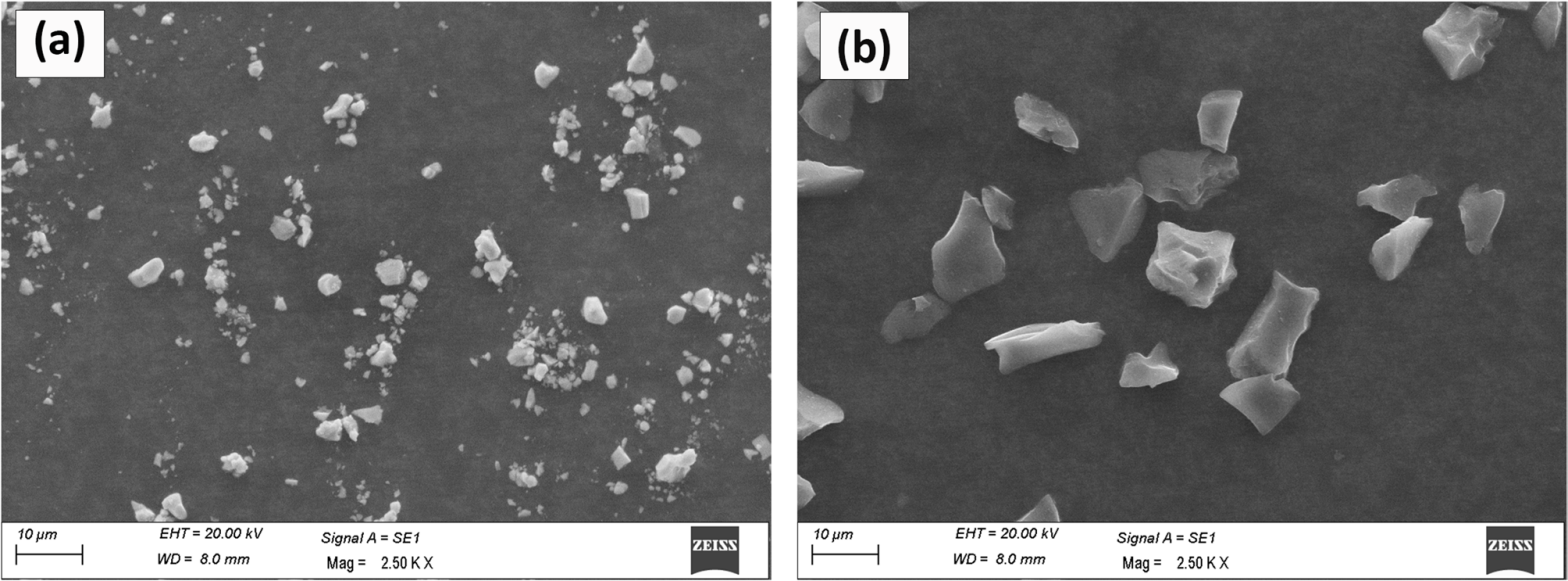
SEM images of reinforcements (a) ZrC particles, (b) SiC particles used in the present study.

The materials used in this study include commercial AA7075 aluminium alloy powder (average particle size approximately 45 μm) as the matrix, along with high-purity silicon carbide (SiC, < 5 μm, 99.5% purity) and zirconium carbide (ZrC, < 4 μm, 99% purity) powders as reinforcements, as shown in Fig. 1. The powders were measured in precise quantities to achieve targeted reinforcement weight fractions, typically ranging from 0 to 9 wt% for SiC and 1–5 wt% for ZrC. Homogeneous dispersion of the reinforcements was accomplished through high-energy ball milling for 2 h at 300 rpm, using a ball-to-powder weight ratio of 10:1 with hardened steel balls.

Following blending, the composite powders were compacted into cylindrical green pellets (20 mm diameter × 5 mm thickness) using a uniaxial hydraulic press at compaction pressures between 300 and 800 MPa, depending on the experimental condition^32^. The green compacts were then sintered in a laboratory-scale microwave furnace operating at 2.45 GHz, with sintering temperatures varied systematically from 400 °C to 650 °C and sintering times from 90 to 130 min^33,34^. The specimens were placed on a susceptor bed to ensure uniform microwave heating, and temperatures were monitored by an infrared pyrometer. Sintered specimens were cooled to room temperature under ambient conditions.

Post-sintering, samples were prepared by sequential grinding and polishing using emery papers of decreasing grit size and subsequently cleaned in acetone. Density measurements were performed by the Archimedes’ principle with ethanol as the immersion medium, while the theoretical density was computed using the rule of mixtures based on the individual particle densities. Microstructural investigations were carried out using scanning electron microscopy (SEM) for assessment of particle dispersion, grain structure, and interfacial quality. Phase analysis was accomplished using X-ray diffraction (XRD).

**Fig. 2:**
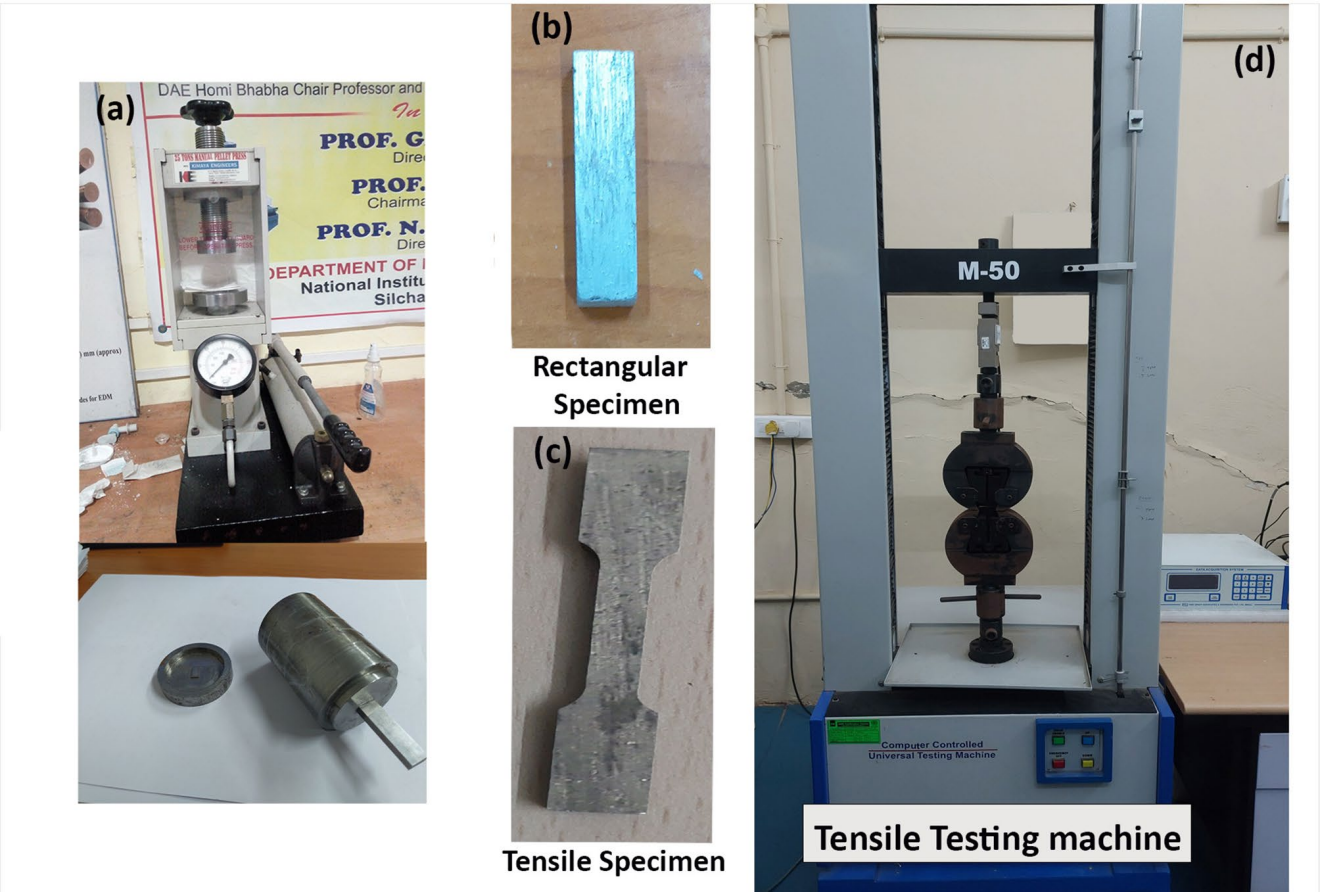
Images showing the equipment (a) manual hydraulic pellet press, (b) rectangular specimen, (c) dog bone shape specimen, (d) Universal Testing Machine (UTM).

Micro Vickers hardness testing was carried out using a load of 100 g and a dwell time of 15 s. For each specimen, five indentations were made at different locations, maintaining a minimum spacing of at least 2–3 times the indentation diagonal length to avoid interaction effects. The reported hardness value corresponds to the average of these measurements, following the standard ASTM E384 procedure. Ultimate tensile strength (UTS) was determined through tensile testing on a universal testing machine at a crosshead speed of 1 mm/min, using dog-bone-shaped specimens machined to conform to ASTM E8 standards. For both microhardness and tensile strength, three specimens were tested for each experimental condition. The reported values correspond to the mean of these measurements, and the associated standard deviation was calculated to quantify experimental variability and ensure measurement reliability. To further assess the reliability of the experimental data, statistical variability in the measured properties was evaluated using standard deviation. The observed variations were within acceptable limits, indicating good repeatability of the fabrication and testing procedures. Minor deviations can be attributed to inherent material heterogeneity, slight variations in reinforcement distribution, and experimental uncertainties associated with measurement techniques. These results confirm that the dataset used for machine learning modeling is statistically consistent and reliable. The equipment’s used to fabricate and testing’s were shown in Fig. 2.

### Data collection and visualization

Prior to model development, the dataset was carefully pre-processed to ensure consistency, including outlier assessment and feature standardization. It is important to note that the present study is intentionally designed to reduce the extensive experimental burden associated with hybrid composite fabrication. In this regard, the dataset comprising 172 samples represents a systematically generated and well-distributed experimental space, capturing the combined influence of key compositional and processing parameters. While larger datasets are generally advantageous in machine learning, their acquisition in materials processing studies is often constrained by significant experimental cost, time, and resource requirements. Therefore, the focus of this work is not on dataset expansion alone, but on developing robust, accurate, and generalizable predictive models using an optimally sized dataset. To ensure reliability, advanced validation strategies such as nested cross-validation, along with rigorous hyperparameter tuning and multi-model comparison, were employed.

A comprehensive set of 172 experimental data points was systematically obtained, each corresponding to unique combinations of compaction pressure, reinforcement weight fractions (SiC & ZrC), sintering temperature, and sintering time. To facilitate initial data exploration and enable insight into parameter-property relationships, a variety of data visualization methods were employed. These included box plots and violin plots to reveal data distributions, contour plots for two-dimensional property mapping, parallel coordinates plots for simultaneously depicting multivariate relationships, hexbin plots to illustrate data point density, and a stream-like plot representing the cumulative distribution of input parameters, aiding visualization of how processing variables are distributed across the experimental space. These visual tools, together with a detailed discussion included in subsequent sections, supported interpretation of dataset coverage and informed the direction of machine learning analyses.

Prior to modeling, the data were pre-processed to remove outliers and standardized. Regression-based machine learning algorithms—namely extreme gradient boosting (XGBoost), random forest, support vector regression (SVR), and artificial neural networks (ANN)—were trained to predict microhardness and tensile strength, evaluated via nested cross-validation to ensure generalizability and robustness. Feature importance analysis revealed the dominant processing parameters affecting the targeted mechanical properties, and final models were validated against hold-out data. This integration of systematic powder metallurgy processing, comprehensive data collection, advanced visualization (including the stream-like cumulative distribution plots), and state-of-the-art machine learning established a robust framework for predictive modeling of microwave-sintered AA7075/SiC/ZrC hybrid composites.

## Results and discussions

### Microstructural analysis

**Fig. 3:**
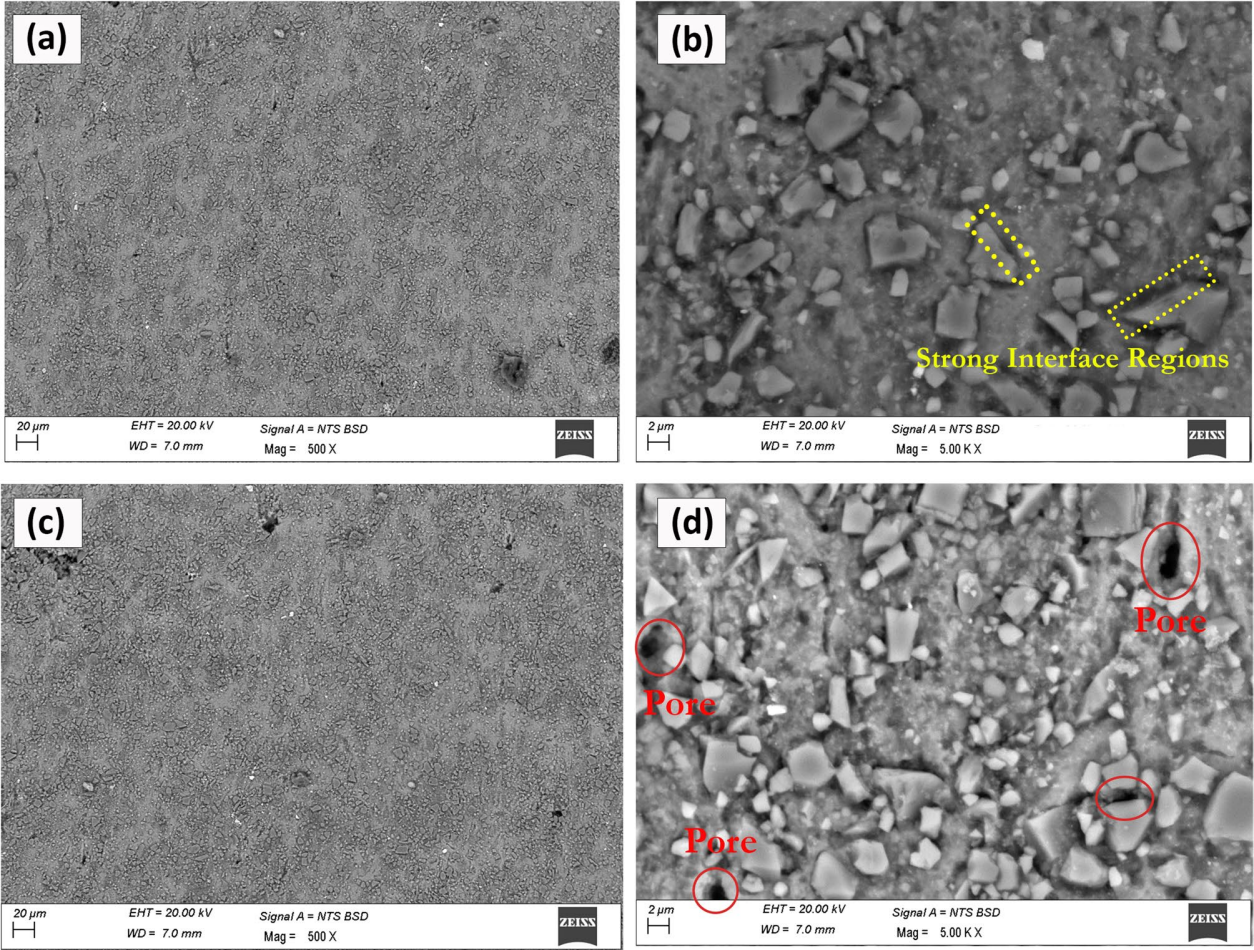
SEM images of AA7075/SiC composite showing (a) SEM micrograph of AA7075 + 7 wt% SiC composite fabricated by conventional powder metallurgy; (b) corresponding EDS spectrum; (c–d) elemental mapping images showing the distribution of Al and SiC particles. The results confirm uniform dispersion of SiC reinforcement within the matrix.

Figure 3 displays SEM images of AA7075/SiC composites with varying SiC contents, providing insights into the dispersion of reinforcements, interfacial characteristics, and the presence of microstructural defects. At a lower magnification (Fig. 3a), the 7% SiC-reinforced composite exhibits a uniform and homogeneous distribution of SiC particles throughout the aluminium matrix, which is essential for efficient load transfer and improved composite performance. The high-magnification image (Fig. 3b) elucidates well-bonded interface regions (highlighted), suggesting robust interactions between the SiC particulates and the matrix. Such strong interfaces are critical, as they promote effective stress transfer under loading, which has been widely reported to enhance the mechanical properties of metal matrix composites (MMCs) by delaying crack initiation and propagation^35,36^. As the SiC content is increased to 9% (Fig. 3c and d), the microstructure reveals the appearance of pits and pores—defects that typically originate due to incomplete sintering, non-uniform consolidation, or local particle clustering. The highlighted pores in Fig. 3d can act as potential stress concentrators, leading to reduced ductility and possibly initiating premature failure during mechanical loading. These observations are consistent with recent MMC studies, which demonstrate that optimal reinforcement dispersion and minimal porosity are key to improving composite hardness and strength, while excessive reinforcement loading may induce microstructural defects and compromise performance^37,38^.

**Fig. 4:**
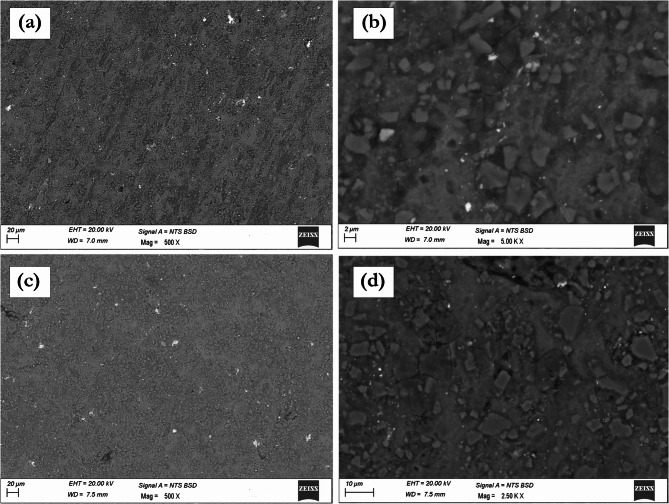
SEM images of AA7075/SiC/ZrC hybrid composite showing (a) uniform dispersion of 6%SiC/2%ZrC reinforcements in the matrix material, (b) high magnification image showing strong interfaces between SiC and ZrC reinforcements with matrix material, (c) uniform dispersion of 7%SiC/3%ZrC reinforcements in the matrix material, (d) high magnification image showing clean interfaces between matrix and SiC and ZrC reinforcements.

Figure 4 presents the SEM characterization of AA7075/SiC/ZrC hybrid composites at different reinforcement fractions. At low magnification, Fig. 4a and c evidence a uniform and homogeneous dispersion of hybrid reinforcements (6% SiC/2% ZrC and 7% SiC/3% ZrC, respectively) within the AA7075 matrix. Such a uniform distribution is advantageous as it impedes dislocation motion more effectively across the entire matrix volume, a primary contributor to the Orowan strengthening mechanism. High-magnification images (Fig. 4b and d) demonstrate clean and well-defined interfaces between the aluminium matrix and both types of reinforcements^39,40^. The hybrid composite interfaces appear smooth with negligible reaction product formation, suggesting favorable wettability and interfacial bonding during microwave sintering. Strong interfaces facilitate direct load transfer from the ductile matrix to the hard ceramic phases, as well as efficient grain boundary pinning, which restricts grain growth and results in grain refinement—a widely reported source of Hall-Petch strengthening in sintered aluminium composites. The absence of agglomeration and porosity in these regions further supports a microstructure optimized for improved hardness and tensile performance, as concurred by recent literature on hybrid particulate composites where synergistic effects from multiple reinforcements yield superior property profiles versus single-phase MMCs^41,42^.

**Fig. 5:**
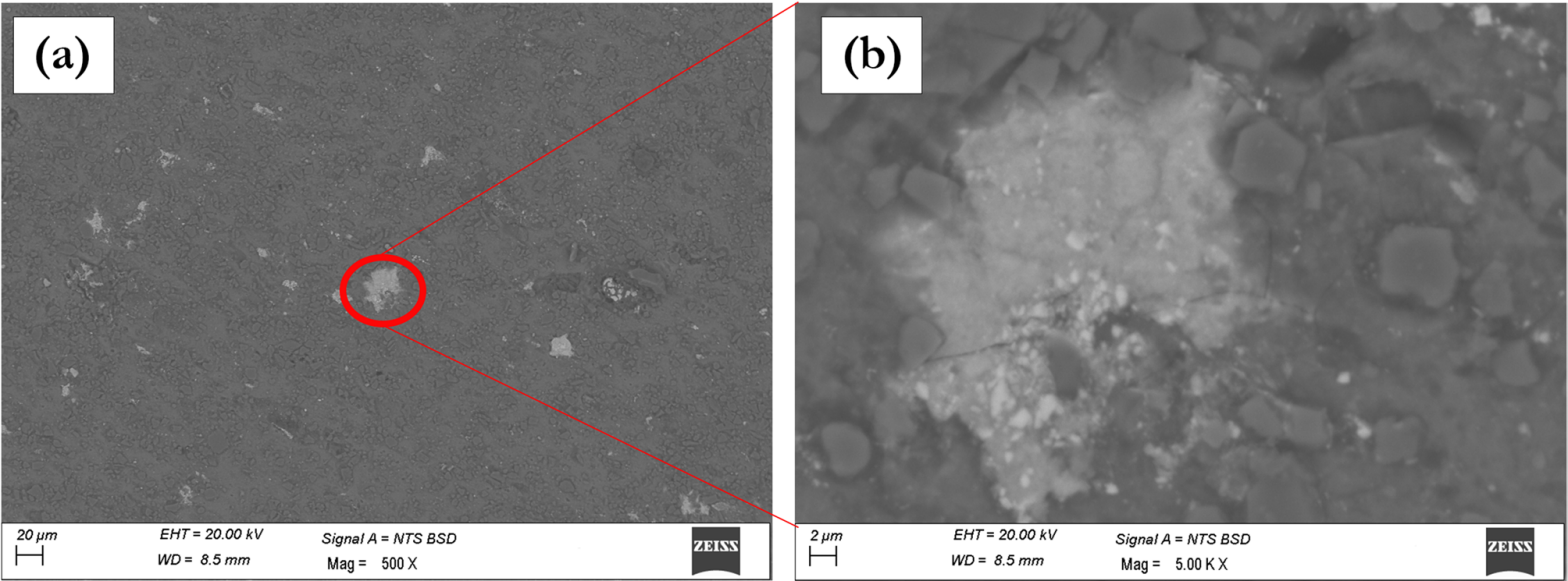
SEM images showing (a) agglomerations of 4% ZrC particles in AA7075/7%SiC hybrid composite, (b) high magnification image showing agglomerate region of ZrC.

Figure 5 documents the effects of further increasing ZrC content in AA7075/SiC/ZrC hybrids. Notably, the SEM observations indicate that agglomerations of ZrC particles begin to appear as the ZrC content reaches 4% and above. Figure 5a clearly shows regions of concentrated ZrC clusters in an AA7075/7%SiC/4%ZrC hybrid, with the agglomerated zone highlighted for emphasis. Figure 5b, a higher magnification view, further elucidates the internal structure of these agglomerates. Such regions arise due to the increasing difficulty in uniformly distributing higher concentrations of ZrC, leading to poor interfacial bonding and localized stress concentration. The microstructural inhomogeneity associated with these agglomerates has been recognized in recent hybrid and nanocomposite research as a detrimental factor—agglomerated particles serve as crack initiation sites under load, undermining the improvements otherwise realized through hybrid reinforcement^6,43^. Thus, it is clear from the present microstructural analysis that the onset of ZrC agglomeration begins from 4% ZrC, with increasing severity at higher loadings, as detailed in Fig. 5.

The mechanical behavior of the hybrid composites is strongly governed by the combined influence of reinforcement distribution, processing conditions, and composition. A uniform dispersion of SiC and ZrC reinforcements within the aluminium matrix facilitates effective load transfer and restricts dislocation motion, thereby enhancing both tensile strength and microhardness. Well-bonded interfaces further contribute to improved stress transfer and structural integrity. In contrast, non-uniform distribution or agglomeration of reinforcements—particularly at higher ZrC contents—introduces localized stress concentrations and porosity, leading to a deterioration in mechanical properties.

Sintering temperature plays a critical role in controlling diffusion, densification, and interfacial bonding. Optimal sintering temperatures promote strong metallurgical bonding and reduced porosity, resulting in improved strength and hardness. However, excessively high temperatures may lead to grain coarsening and degradation of interfacial integrity, which negatively impacts mechanical performance.

Composition also significantly influences the observed properties. Increasing SiC content enhances hardness and strength due to its inherent stiffness and strengthening mechanisms such as Orowan strengthening and grain refinement. Similarly, moderate addition of ZrC improves interfacial stability and high-temperature performance. However, excessive reinforcement content leads to agglomeration and defects, offsetting these benefits. These observations highlight the importance of achieving an optimal balance between reinforcement distribution, composition, and processing conditions to maximize mechanical performance^44^.

In summary, SEM analyses (Figs. 3, 4 and 5) demonstrate that the uniform dispersion of SiC and ZrC particulates and the formation of strong matrix/reinforcement interfaces are essential for optimizing the mechanical properties of microwave-sintered AA7075 composites. Uniformly distributed, well-bonded reinforcements up to 3% ZrC contribute to enhanced microhardness and tensile strength through Orowan strengthening, grain refinement, and effective load transfer. However, beginning at 4% ZrC (Fig. 3), agglomeration emerges as a significant microstructural defect, detracting from composite performance. These results underscore the critical balance between reinforcement content and microstructural control, corroborating recent literature findings and reinforcing the importance of meticulous process optimization in high-performance aluminium matrix hybrid composites.

### Mechanical properties

**Fig. 6:**
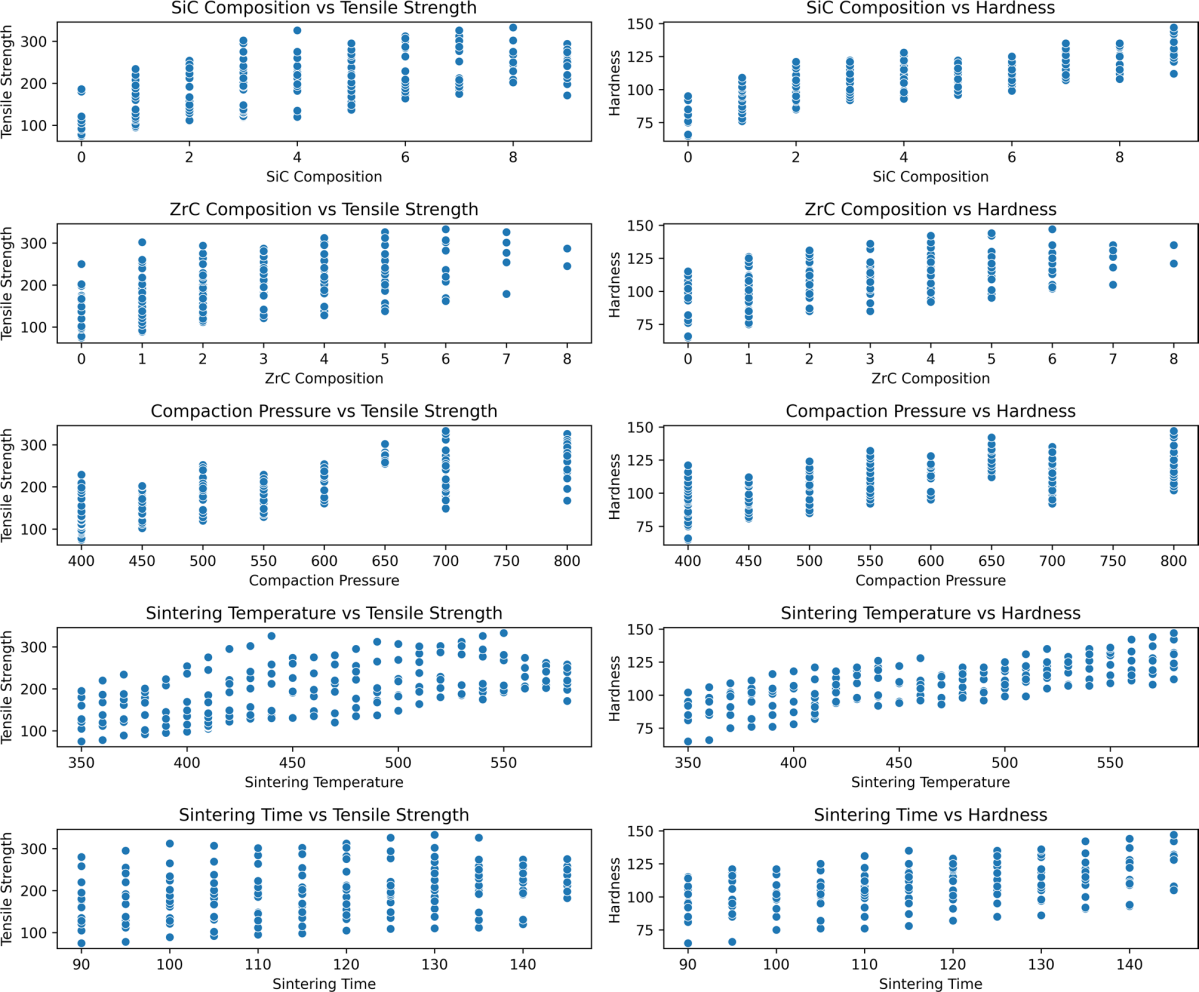
Correlations of tensile strength and hardness with processing parameters: (a) ZrC composition, (b) compaction pressure, (c) sintering temperature, and (d) sintering time.

Figure 6 illustrates the relationships between the key mechanical properties—tensile strength and microhardness—and the principal processing variables: SiC composition, ZrC composition, compaction pressure, sintering temperature, and sintering time. The observed trends provide valuable insights into how compositional and processing parameters influence the microstructure and performance of microwave-sintered AA7075/SiC/ZrC hybrid composites^45^.

An initial increase in SiC content results in notable improvements in both tensile strength and hardness. This enhancement is chiefly attributed to several strengthening mechanisms introduced by finely dispersed SiC particles. Firstly, SiC’s inherent hardness and compatibility with the aluminium matrix facilitate efficient load transfer from matrix to reinforcement. Secondly, SiC impedes dislocation motion through Orowan strengthening, thereby increasing resistance to plastic deformation^46^. Additionally, SiC particles act as heterogeneous nucleation sites during sintering, promoting grain refinement and further enhancing strength, consistent with the Hall–Petch relationship. However, beyond an optimal content—commonly observed around 8–9 wt%—the benefits plateau or slightly decline. SEM analyses from earlier sections reveal that higher SiC concentrations lead to agglomeration and occasional pore formation, generating local stress concentrations and compromised interfaces. These defects serve as crack initiation points, ultimately restricting further gains in mechanical performance^47^. These findings align with existing literature, which consistently identifies an optimal SiC threshold for maximizing property improvements without inducing microstructural weaknesses.

The effect of ZrC addition closely parallels that of SiC: moderate ZrC contents (up to ~ 3 wt%) further improve tensile strength and hardness due to synergistic reinforcement. ZrC offers excellent wettability with aluminium and fosters strong interfaces, enabling effective stress transfer and improved microstructural stability. The combination of SiC and ZrC also leverages the unique properties of both phases—hardness from SiC and high-temperature stability from ZrC—leading to superior composite performance. When ZrC content surpasses approximately 3–4 wt%, a decline in properties is observed. This trend can be directly correlated to the SEM observations (as shown in Figs. 4 and 5), where excessive ZrC results in noticeable agglomeration and non-uniformity within the matrix. These agglomerates disrupt load transfer pathways and introduce microvoids, thus diminishing both tensile and hardness properties. Such behavior highlights the necessity of maintaining optimal reinforcement levels to prevent clustering and maximize hybrid synergy^48,49^.

Increasing compaction pressure generally contributes to higher tensile strength and microhardness up to an optimum value. Enhanced compaction promotes better powder particle packing, reduces initial porosity, and improves green density—factors that positively affect the densification and interfacial integrity during sintering. These improvements yield a more continuous load-bearing matrix and superior mechanical properties. Nevertheless, excessively high compaction pressure may introduce work hardening or induce microcracks in the green compacts, which can propagate during sintering, but within the experimental range studied, the overall influence was beneficial with no detrimental effects observed^50,51^.

The influence of sintering temperature exhibits a classic bell-shape trend: increasing temperature initially enhances both tensile strength and hardness, attributed to improved diffusion, particle bonding, and overall densification. Higher sintering temperatures facilitate better metallurgical bonding at the matrix–reinforcement interfaces, reducing residual porosity. However, when the temperature exceeds an optimal point, grain coarsening and the potential for localized melting or interfacial degradation emerge^52^. These phenomena reduce grain boundary area and impair the fine dispersion of reinforcements, resulting in diminished mechanical properties.

Sintering time affects the extent of densification and grain growth. Moderate increases in dwell time yield better particle bonding, decreased porosity, and enhanced mechanical response. Yet, prolonged exposure initiates grain growth and possible deleterious intermetallic phase formation, particularly at the interface, which then adversely impacts both strength and hardness. Thus, an optimal sintering time balances the competing needs of diffusion-driven bonding and grain size control^53^.

In summary, both SiC and ZrC compositions play pivotal roles in enhancing tensile and hardness properties through combined mechanisms such as load transfer, Orowan strengthening, grain refinement, and strong matrix–reinforcement interfaces^54^. However, exceeding optimal reinforcement levels introduces agglomeration and microstructural defects, negating these benefits. Similarly, compaction pressure, sintering temperature, and sintering time must each be optimized to maximize density and interfacial integrity without inducing excessive grain coarsening or porosity. These collective findings are congruent with recent advanced studies on hybrid aluminium matrix composites, emphasizing the need for a carefully balanced process–composition window to achieve superior mechanical performance^55,56^.

Table 1 presents a comparative overview of previously reported Al-based and AA7075-based hybrid composites reinforced with SiC and secondary ceramic phases. It is evident that the mechanical performance of these composites is strongly influenced by both the type of reinforcement and the processing route. For instance, conventional casting routes often result in inferior or inconsistent properties due to porosity, particle clustering, and elemental losses, as observed in AlSi9Mg–SiC composites^57^. Similarly, although hybrid reinforcements such as SiC–Gr and SiC–ZrO₂ enhance hardness and strength, their effectiveness is limited by processing-induced defects in casting-based methods^58,59^.

In contrast, advanced processing techniques such as microwave sintering significantly improve the interfacial bonding, densification, and overall mechanical performance of AA7075-based composites. Previous studies on AA7075–SiC–ZrC composites demonstrated a tensile strength of up to ~ 461 MPa^60^, while a systematic comparison of fabrication routes reported a tensile strength of 321 MPa for microwave-processed composites, emphasizing the critical role of processing conditions^61–63^.

Building upon these findings, the present work introduces a data-driven approach by integrating powder metallurgy with machine learning models (ANN and XGBoost) to predict mechanical properties with high accuracy. Unlike traditional trial-and-error methods, the proposed approach enables efficient prediction and optimization of composite performance, thereby offering a scalable and intelligent framework for advanced composite design^64,65^.

**Table 1:** Comparison of mechanical properties and processing routes of Al-based and AA7075-based hybrid composites reinforced with SiC and secondary ceramic phases.

Ref.	Material system	Reinforcement	Processing method	Tensile strength (MPa)	Hardness	Key findings
^57^	AlSi9Mg–20%SiC	SiC (20%)	Conventional casting + remelting	310 → 128	~ 105 HBW	Severe degradation due to porosity, Mg loss, clustering
^58^	Al6061–SiC–Gr	SiC (13%) + Gr (1–3%)	Stir casting + cryogenic	~ 145	~ 146–150 VHN	Cryogenic treatment improves strength & hardness
^59^	Al–SiC / Al–ZrO_2_–SiC	SiC, ZrO_2_	Casting	67 → 96	~ 50 h	Hybrid reinforcement improves hardness & strength
^60^	AA7075–SiC–ZrC	SiC + ZrC	Microwave sintering	~ 461	~ 122 HV	Hybrid reinforcement + microwave improves properties
^61^	AA7075–SiC–ZrC	SiC + ZrC	Stir casting / PM / Microwave	321	~ 136 HV	Fabrication technique strongly influences performance; microwave sintering gives best results
Present work	AA7075–SiC–ZrC	SiC + ZrC	Powder metallurgy + ML	(Predicted high accuracy)	(Predicted)	ML models (ANN, XGBoost) accurately predict properties

The table highlights the influence of fabrication techniques, reinforcement combinations, and advanced processing approaches, along with the role of machine learning in property prediction.

### Data analysis and visualization

Prior to engaging machine learning algorithms for predictive modelling, a comprehensive visual exploration of the experimental dataset is indispensable. Data visualization acts as an essential intermediary between raw experimental data and actionable scientific understanding, allowing researchers to intuitively grasp the underlying structure, distribution, and variability present within complex, multidimensional data. These graphical tools facilitate the detection of trends, highlight the presence of outliers or anomalies, identify potential correlations among variables, and ensure that the collected dataset provides adequate coverage across the experimental design space. By illuminating these critical factors in advance, data visualization assists in data pre-processing, guides feature selection, and ensures a more robust and interpretable modelling process.

**Fig. 7:**
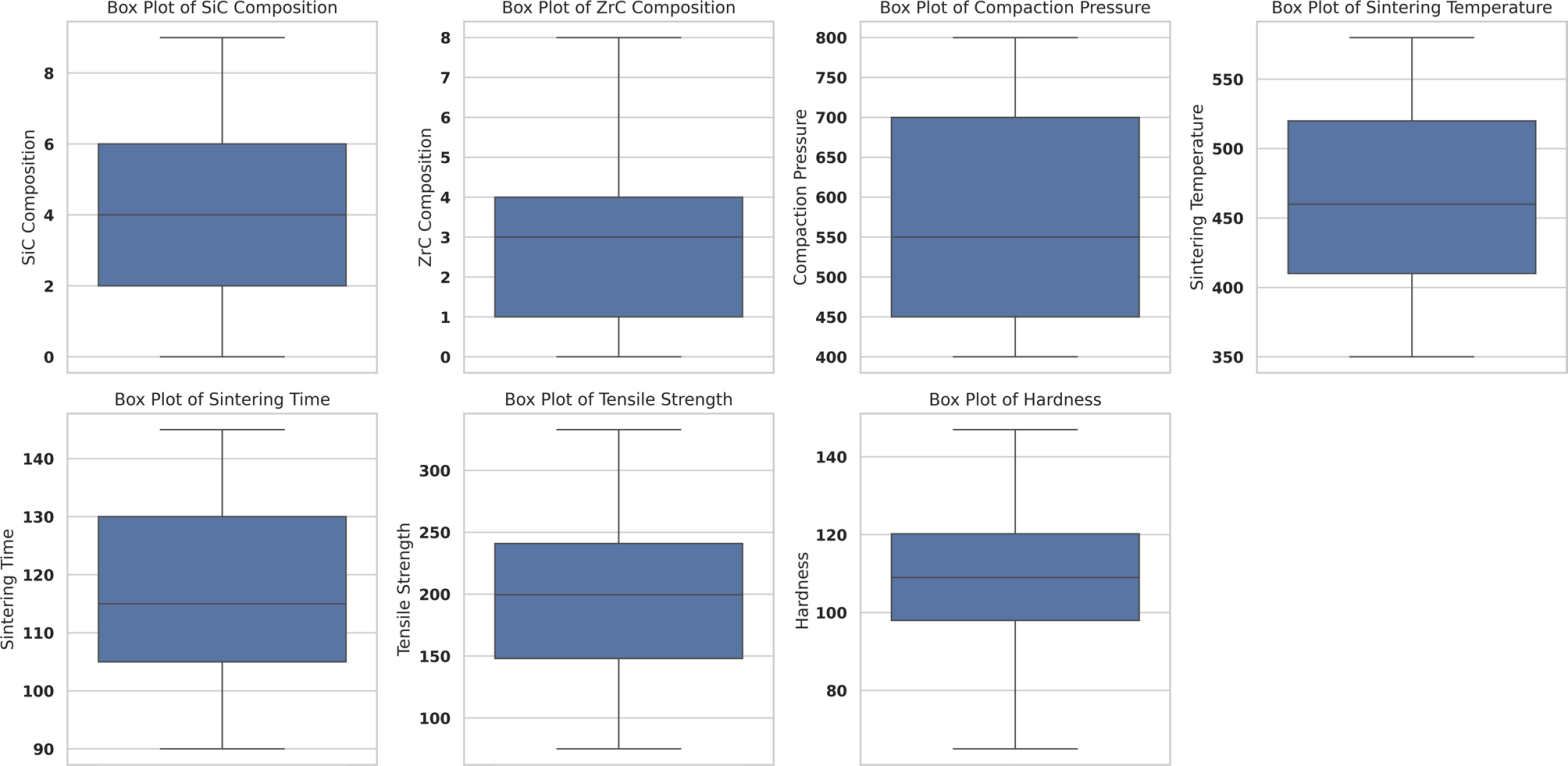
Box plots illustrating the distribution of key input and output parameters used in the study, including SiC Composition, ZrC Composition, Compaction Pressure, Sintering Temperature, Sintering Time, Tensile Strength, and Hardness. These plots help visualize the spread, central tendency, and presence of any outliers in the dataset.

In this study, a suite of advanced visualization techniques was employed to systematically examine both input and output variables. Box plots (Fig. 7) were utilized to succinctly summarize the spread, central tendency, and potential outliers across all major parameters, including SiC composition, ZrC composition, compaction pressure, sintering temperature, sintering time, tensile strength, and hardness. The majority of these variables exhibited broad, well-balanced distributions, reflecting effective experimental sampling. The output parameters also displayed substantial variability, which is crucial for training predictive models with strong generalizability. The absence of extreme outliers further attests to the consistency and reliability of the experimental methodology^66^.

**Fig. 8:**
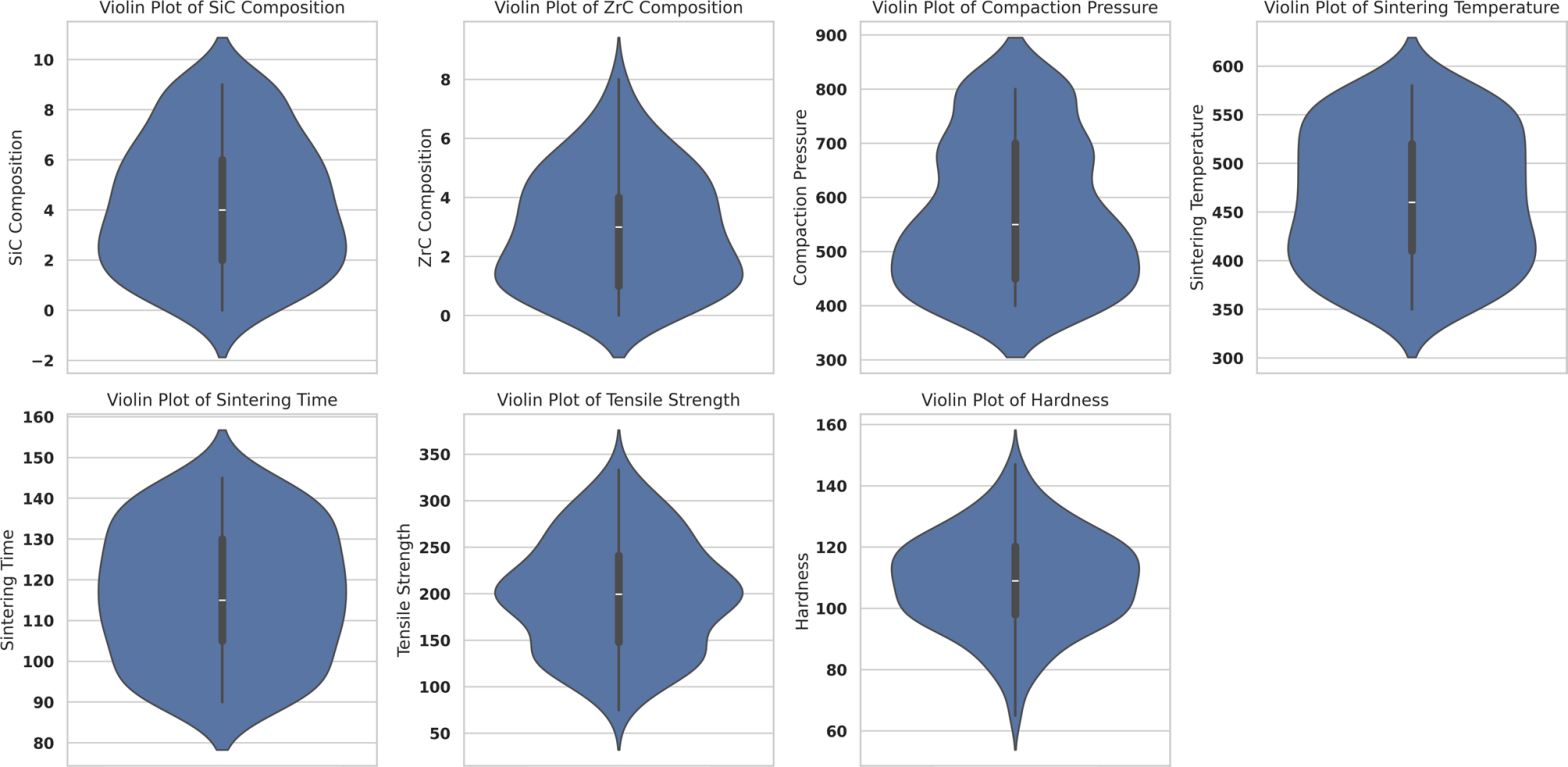
Violin plots showing the distribution and probability density of input parameters (SiC Composition, ZrC Composition, Compaction Pressure, Sintering Temperature, and Sintering Time) and output parameters (Tensile Strength and Hardness). These plots provide insights into the data distribution, highlighting concentration zones and potential multimodal patterns across each parameter.

To delve deeper into the shape and density of data distributions, violin plots (Fig. 8) were deployed. These visualizations offered a richer perspective than box plots alone, as they rendered the underlying probability densities of each parameter. Clear trends, such as unimodal and occasionally multimodal distributions, were detected, indicating that some parameters had clustered regions, possibly due to batch-specific optimization runs. For reinforcement contents, the data were concentrated around mid-range values, yet adequately spanned the full experimental range, ensuring sufficient data diversity for capturing complex, nonlinear relationships. Similar smooth, single-peaked densities in the output variables suggested that mechanical properties such as tensile strength and hardness varied in a systematic and physically meaningful fashion with respect to the input processing conditions^67^.

**Fig. 9:**
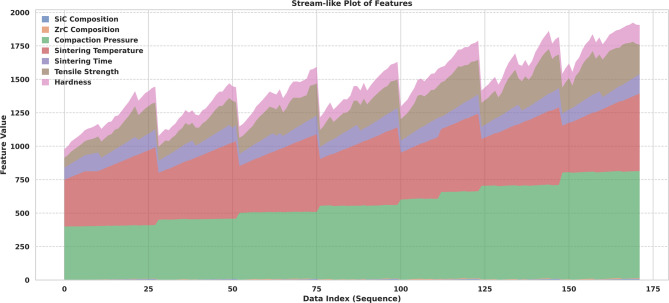
Stream-like plot representing the cumulative distribution of input parameters (SiC Composition, ZrC Composition, Compaction Pressure, Sintering Temperature, Sintering Time) and output parameters (Tensile Strength and Hardness) across the dataset. This visualization highlights how feature values evolve sequentially, allowing observation of trends, groupings, or gradual variations in parameter magnitudes.

A novel stream-like plot (Fig. 9) was incorporated to visualize the cumulative distribution of both input and output parameters across the entire dataset. This approach enabled clear tracking of how features collectively evolved over the course of the experimental campaign, revealing sequential patterns, groupings, and the interdependence between process variables and mechanical response. Such visualization allowed for easy identification of experimental “waves” or batches where particular parameters were systematically varied, providing insights into potential interaction effects or experimental design strategies^68^.

**Fig. 10:**
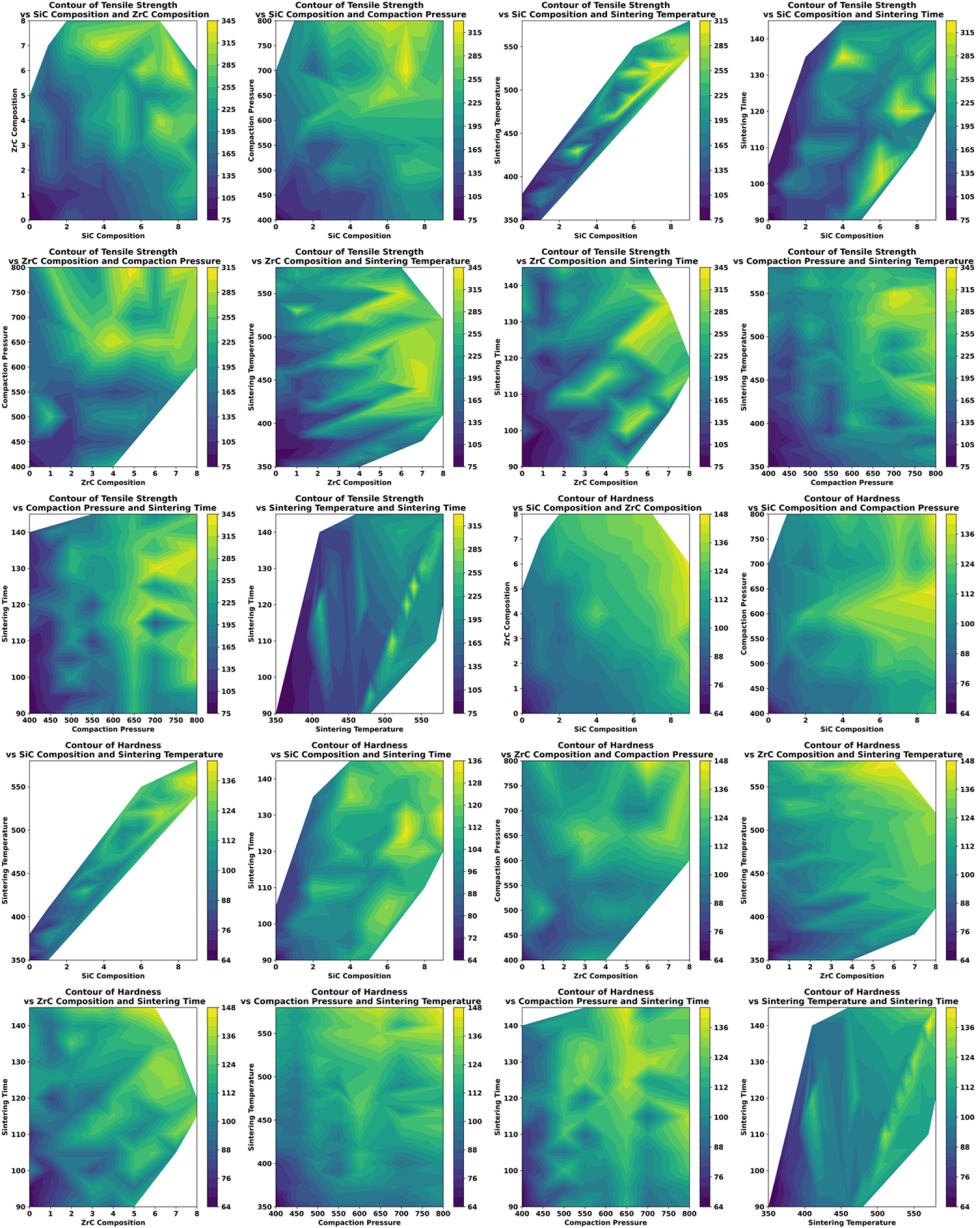
Contour plots depicting the influence of input parameters—SiC Composition, ZrC Composition, Compaction Pressure, Sintering Temperature, and Sintering Time—on the output responses: Tensile Strength (top three rows) and Hardness (bottom three rows). Each subplot illustrates the Interaction between two parameters while the remaining are held constant, enabling identification of synergistic or antagonistic effects on mechanical properties. Warmer colors indicate higher values of tensile strength or hardness, aiding in optimal parameter region identification.

To further unravel the complex, multivariate interactions affecting mechanical performance, contour plots (Fig. 10) were generated for both tensile strength and hardness. By graphically mapping the simultaneous impact of two parameters at a time—while keeping the others constant—these plots illuminated zones of synergistic optimization and regions susceptible to antagonistic effects such as reinforcement agglomeration or excessive grain growth. The use of warm color gradients helped to readily identify optimal parameter spaces, highlighting processing and compositional windows associated with peak mechanical performance. Conversely, cooler regions indicated parameter combinations where property enhancements plateaued or declined, often corresponding to microstructural defects or non-ideal consolidation.

**Fig. 11:**
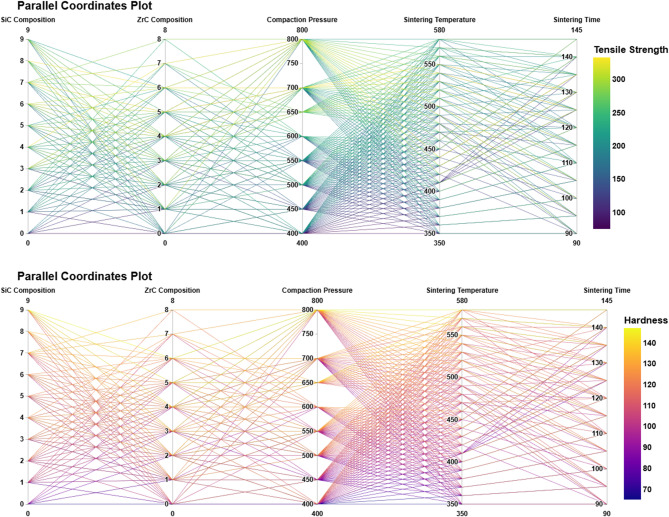
Parallel coordinates plots illustrating the variation in mechanical properties—Tensile Strength (top) and Hardness (bottom)—with respect to the process parameters: SiC Composition, ZrC Composition, Compaction Pressure, Sintering Temperature, and Sintering Time. Each line represents one data instance, and the color gradient corresponds to the respective output value. These plots facilitate the identification of parameter combinations leading to optimal mechanical performance.

**Fig. 12:**
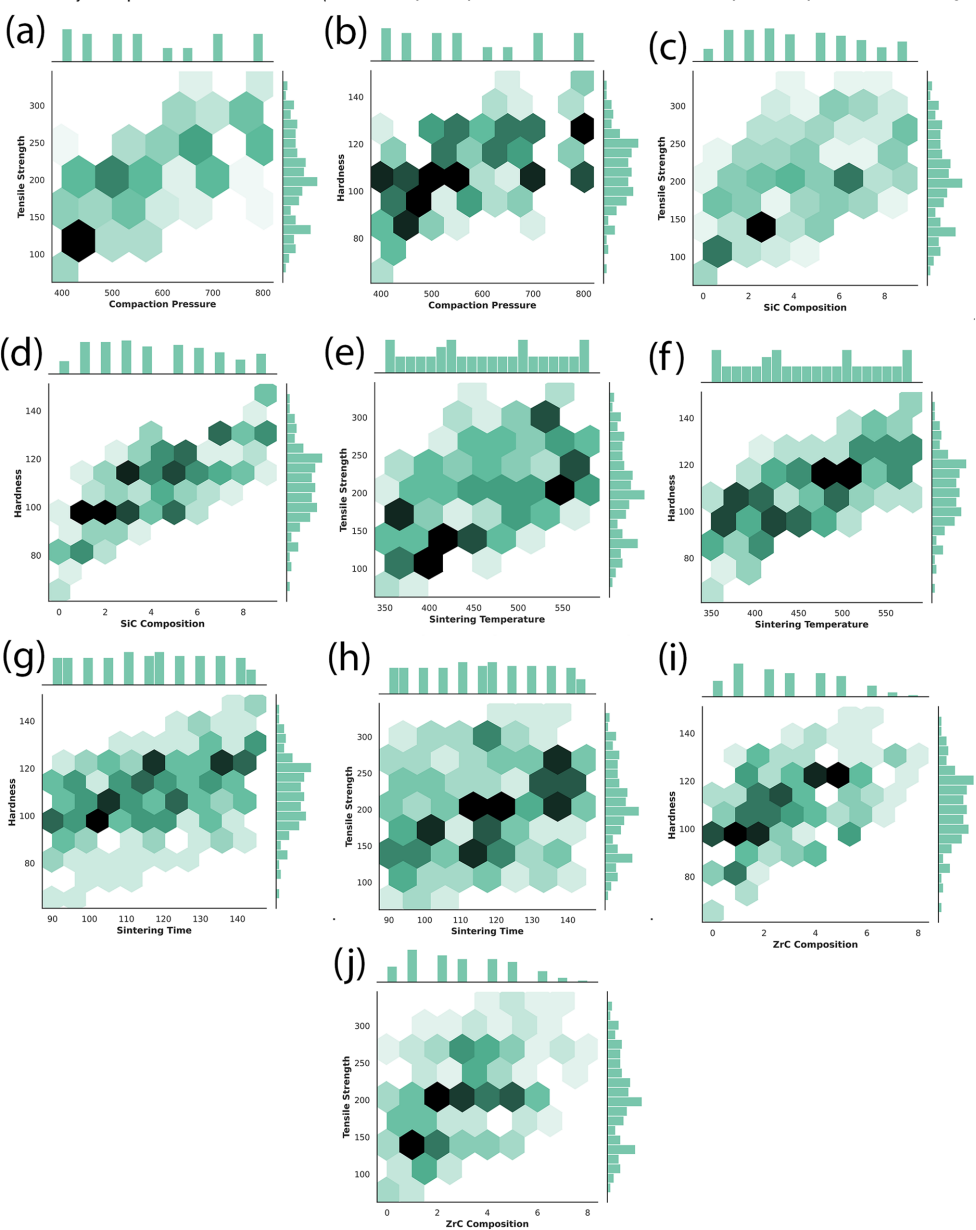
Hexbin plots showing the relationship between mechanical properties and individual process parameters. (a), (c), (e), (h), (j) illustrate the effect of Compaction Pressure, SiC Composition, Sintering Temperature, Sintering Time, and ZrC Composition on Tensile Strength, respectively. (b), (d), (f), (g), (i) show corresponding relationships with Hardness. Darker hexagons represent higher data point densities. These plots highlight significant trends and correlations, such as increasing tensile strength and hardness with higher SiC composition, compaction pressure, and sintering temperature.

Building on the insights established from earlier visualizations, which clarified the spread, central tendency, and distribution shapes of both the input parameters and mechanical properties, Figs. 11 and 12 advance the analysis by capturing how these variables interact in higher-dimensional and pairwise relationship spaces. The parallel coordinates plots in Fig. 11, for both tensile strength and hardness, offer a holistic depiction of the dataset’s complexity: each line traces a complete experimental instance across all relevant process variables, colored according to the achieved mechanical response. Trends previously suggested by box, violin, and stream-like plots become more explicit here, as lines depicting the highest tensile and hardness values consistently weave through optimal ranges of SiC composition, moderate to high compaction pressures, and favourable windows of sintering temperature and time. These visual intersections underscore not only the importance of individual parameters, but also their synergy—demonstrating how specific parameter combinations repeatedly yield superior performance while others are more commonly associated with diminished outcomes, such as the decline seen at excessive ZrC content or prolonged sintering.

Further complementing these multidimensional portrayals, the hexbin plots in Fig. 12 distill the relationships down to focused, bivariate views, mapping the density and direction of data trends between each mechanical property and individual process variables. As anticipated from the broader distributions, both tensile strength and hardness increase steadily with higher SiC composition and compaction pressure, and reach their peak values within optimal intervals of sintering temperature and time, after which gains taper due to phenomena like particle agglomeration or grain coarsening. The hexagonal density shading reinforces where experimental data is most concentrated, providing confidence in the robustness and reproducibility of the observed trends, such as the strong, nearly monotonic improvement in microhardness with rising SiC content or the critical compaction pressure threshold above which further gains are limited.

In essence, these advanced visualization strategies serve as an integrative conclusion to the suite of data exploration tools deployed prior to machine learning analysis. By mapping not just simple distributions or cumulative profiles, but also intricate interdependencies between variables, they allow for clear recognition of optimal processing routes, provide empirical confirmation for microstructural observations made earlier, and lay a rigorous, intuitive foundation for the subsequent development of predictive models. This stepwise visualization-driven approach ensures that the mechanical property enhancements documented throughout the work are not only statistically sound but also physically interpretable in terms of material design practice for AA7075/SiC/ZrC hybrid composites.

### Evaluation of machine learning models

To develop an accurate and robust predictive framework for estimating the tensile strength and hardness of microwave-sintered AA7075/SiC/ZrC hybrid composites, a variety of state-of-the-art machine learning regression algorithms were systematically explored. These included Extreme Gradient Boosting (XGBoost), Random Forest (RF), Support Vector Regression (SVR), K-Nearest Neighbors (KNN), and Artificial Neural Networks (ANN). To ensure robust and unbiased model evaluation, a nested cross-validation framework was employed. In the outer loop, the dataset was partitioned into k folds (k = 5), where one-fold was used as the test set and the remaining k − 1 folds were used for training. Within each training set, an inner k-fold cross-validation was performed to optimize model hyperparameters. Hyperparameter tuning was carried out using a grid search approach, where combinations of parameters were systematically evaluated to identify the optimal configuration based on validation performance. This nested structure ensures that the test data remain unseen during model tuning, thereby providing a reliable estimate of model generalization. XGBoost achieved optimal prediction with carefully chosen tree depths and subsampling rates, while Random Forest benefited from deep ensemble structures. The KNN model, with k set to three and distance-based weighting, provided strong local interpolation capability, and SVR’s best performance was obtained with a high penalty parameter and a radial basis function kernel. For the ANN, a single hidden layer of 100 neurons using the ‘tanh’ activation function yielded the best balance between expressiveness and stability. The full list of optimized hyperparameters for each target property is detailed in Table 2.

Comparative assessment of model performance, as summarized in Table 3, reveals that all models delivered high predictive accuracy, with R² values well above 0.91 for both tensile strength and hardness. Among them, the ANN model stood out for tensile strength prediction, achieving an R² of 0.9748 and the lowest MAE (7.45) and RMSE (9.59), indicating its strong ability to capture the underlying complexities of the mechanical response. For microhardness prediction, XGBoost performed best with an R² of 0.9595 and the lowest error metrics, demonstrating its robustness for this specific property. KNN and SVR also provided competitive results, particularly excelling in regions where local data density supported strong interpolation or where nonlinear relationships dominated. Random Forest, though highly reliable, showed slightly reduced precision for hardness compared to XGBoost and SVR. Overall, these findings confirm the effectiveness of advanced regression methods for mapping complex, multivariate relationships between processing conditions and mechanical performance in hybrid metal matrix composites. A detailed analysis of model behavior, including the interpretability of learned features and the implications for materials design, is provided in the following sections.

**Table 2:** Optimized hyperparameters for each model used in the study obtained through GridsearchCV technique.

	Tensile strength	Hardness
XG Boost	‘colsample_bytree’: 0.6, ‘learning_rate’: 0.1, ‘max_depth’: 3, ‘n_estimators’: 300, ‘subsample’: 1.0	‘colsample_bytree’: 1.0, ‘learning_rate’: 0.05, ‘max_depth’: 5, ‘n_estimators’: 300, ‘subsample’: 0.6
Random Forest	‘max_depth’: None, ‘min_samples_leaf’: 1, ‘min_samples_split’: 2, ‘n_estimators’: 200	‘max_depth’: 10, ‘min_samples_leaf’: 1, ‘min_samples_split’: 2, ‘n_estimators’: 200
KNN	‘n_neighbors’: 3, ‘p’: 2, ‘weights’: ‘distance’	‘n_neighbors’: 3, ‘p’: 2, ‘weights’: ‘distance’
SVR	‘C’: 100, ‘gamma’: 0.1, ‘kernel’: ‘rbf’	‘C’: 100, ‘gamma’: ‘auto’, ‘kernel’: ‘rbf’
ANN	‘activation’: ‘tanh’, ‘alpha’: 0.0001, ‘hidden_layer_sizes’: (100,), ‘learning_rate’: ‘constant’, ‘solver’: ‘sgd’	‘activation’: ‘tanh’, ‘alpha’: 0.0001, ‘hidden_layer_sizes’: (100,), ‘learning_rate’: ‘constant’, ‘solver’: ‘sgd’

**Table 3:** Performance metrics of the machine learning models used in the study.

	*R* Square		MAE		RMSE	
	Tensile	Hardness	Tensile	Hardness	Tensile	Hardness
Random Forest	0.9575	0.9187	9.2900	3.3495	12.4620	4.1822
XG Boost	0.9581	0.9595	9.6003	2.2958	12.3825	2.9523
KNN	0.9639	0.9163	8.3894	3.2244	11.4901	4.2452
SVR	0.9646	0.9493	8.1428	2.4771	11.3834	3.3048
ANN	0.9748	0.9245	7.4515	3.0818	9.5873	4.0298

#### Random Forest model

The Random Forest (RF) algorithm, employed in this study for predicting the tensile strength and hardness of microwave-sintered AA7075/SiC/ZrC hybrid composites, is an ensemble learning method that constructs a multitude of decision trees during training and outputs the mean prediction of the individual trees for regression tasks. By aggregating the results of numerous decorrelated trees, RF mitigates overfitting common to single decision trees and enhances the robustness and generalizability of predictions. Forest models excel particularly when handling datasets that contain nonlinear relationships, complex feature interactions, and moderate to high dimensionality—conditions typical in material science datasets involving multivariate processing parameters and property responses. Furthermore, RF is capable of managing datasets with noisy or incomplete data, due to its inherent averaging over multiple trees which reduces variance and improves stability.

**Fig. 13:**
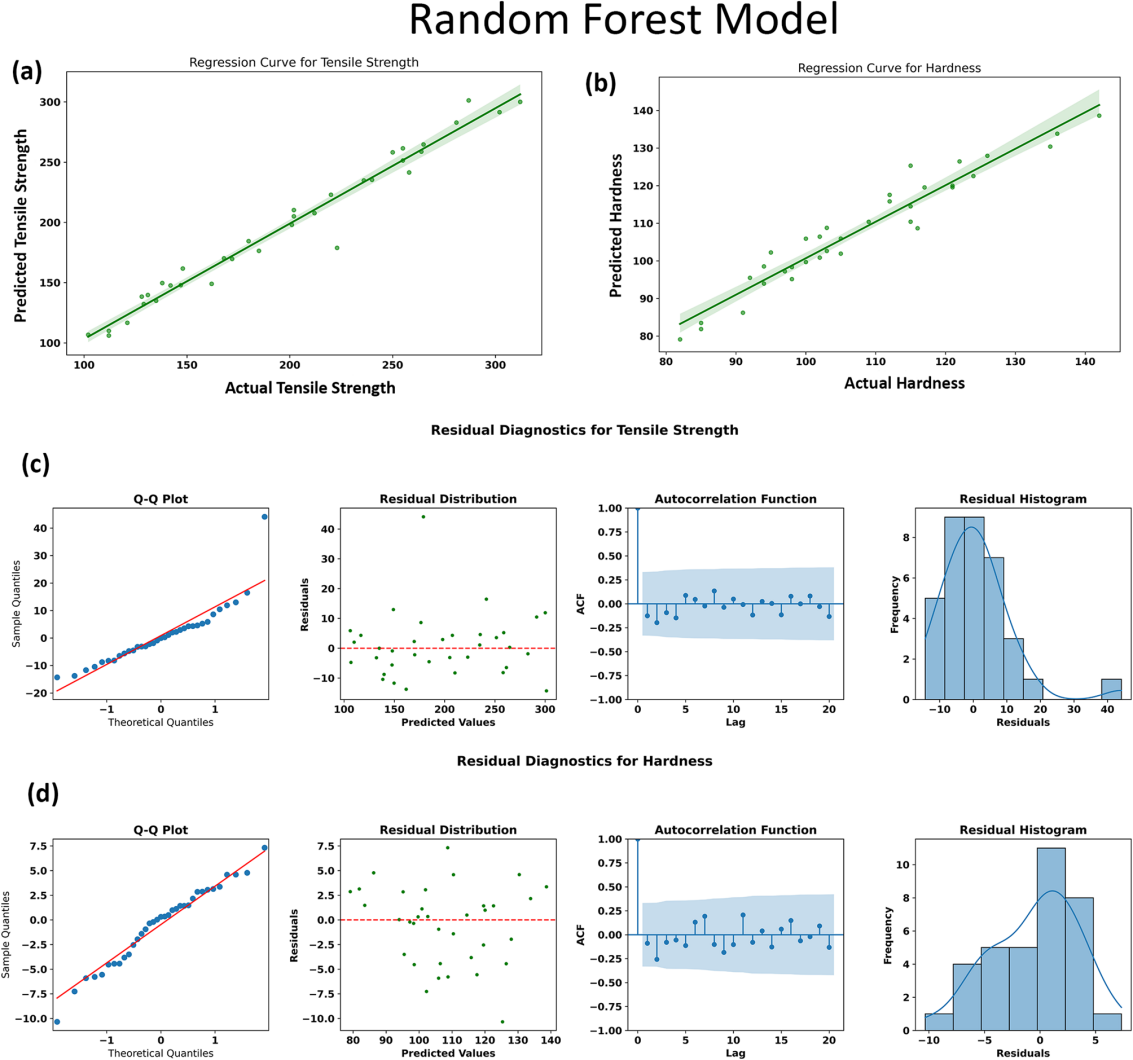
Performance evaluation and residual diagnostics of the Random Forest model for predicting tensile strength and hardness. (a) and (b) show the regression plots comparing actual versus predicted values for tensile strength and hardness, (c) and (d) depict the residual diagnostics for tensile strength and hardness, including Q-Q plots, residual scatter plots, autocorrelation functions, and histograms.

In the context of this investigation, Fig. 13 highlights the effectiveness of the Random Forest model in capturing the tensile strength and hardness trends. The regression plots (Fig. 13a and b) illustrate a strong correlation between the actual measured values and the model predictions, indicating reliable prediction accuracy. This aligns well with the performance metrics reported, where RF achieved R² values of 0.9575 for tensile strength and 0.9187 for hardness, accompanied by relatively low mean absolute error (MAE) and root mean square error (RMSE) values. Such results validate RF’s capacity to model the underlying nonlinear and multivariate dependences in the composite dataset effectively.

Residual diagnostics further reinforce the model’s suitability. The Q-Q plots (Fig. 13c and d) depict a near-linear trend along the diagonal, suggesting that residuals follow a distribution close to normality, which is a desirable trait indicating unbiasedness and homoscedasticity. The scatter plots of residuals versus predicted values exhibit no apparent patterns or heteroscedasticity, pointing to consistent performance across the prediction range. Autocorrelation function plots demonstrate negligible serial correlation in residuals, confirming that errors are independent and random. Lastly, residual histograms show symmetric distributions centered around zero, further affirming the model’s balanced prediction behavior.

Taken together, these diagnostics indicate that the Random Forest model not only fits the data well but also satisfies important statistical assumptions, lending confidence in its predictive validity. Its strong performance with this dataset underscores its suitability for material property prediction problems characterized by moderate data size, nonlinear parameter-property relationships, and multiple interacting variables—as in the case of the AA7075/SiC/ZrC hybrid composite system investigated here.

#### XG Boost

Extreme Gradient Boosting (XGBoost) is a powerful ensemble learning technique that utilizes an optimized implementation of gradient-boosted decision trees. Unlike traditional random forests which average predictions from independently grown trees, XGBoost builds trees sequentially, with each new tree aiming to correct the errors made by the ensemble thus far. This iterative process, combined with efficient regularization, shrinkage, and robust handling of missing data, enables XGBoost to capture intricate nonlinear relationships and complex feature interactions, making it exceptionally suitable for high-dimensional, heterogeneous datasets often encountered in advanced materials science research.

**Fig. 14:**
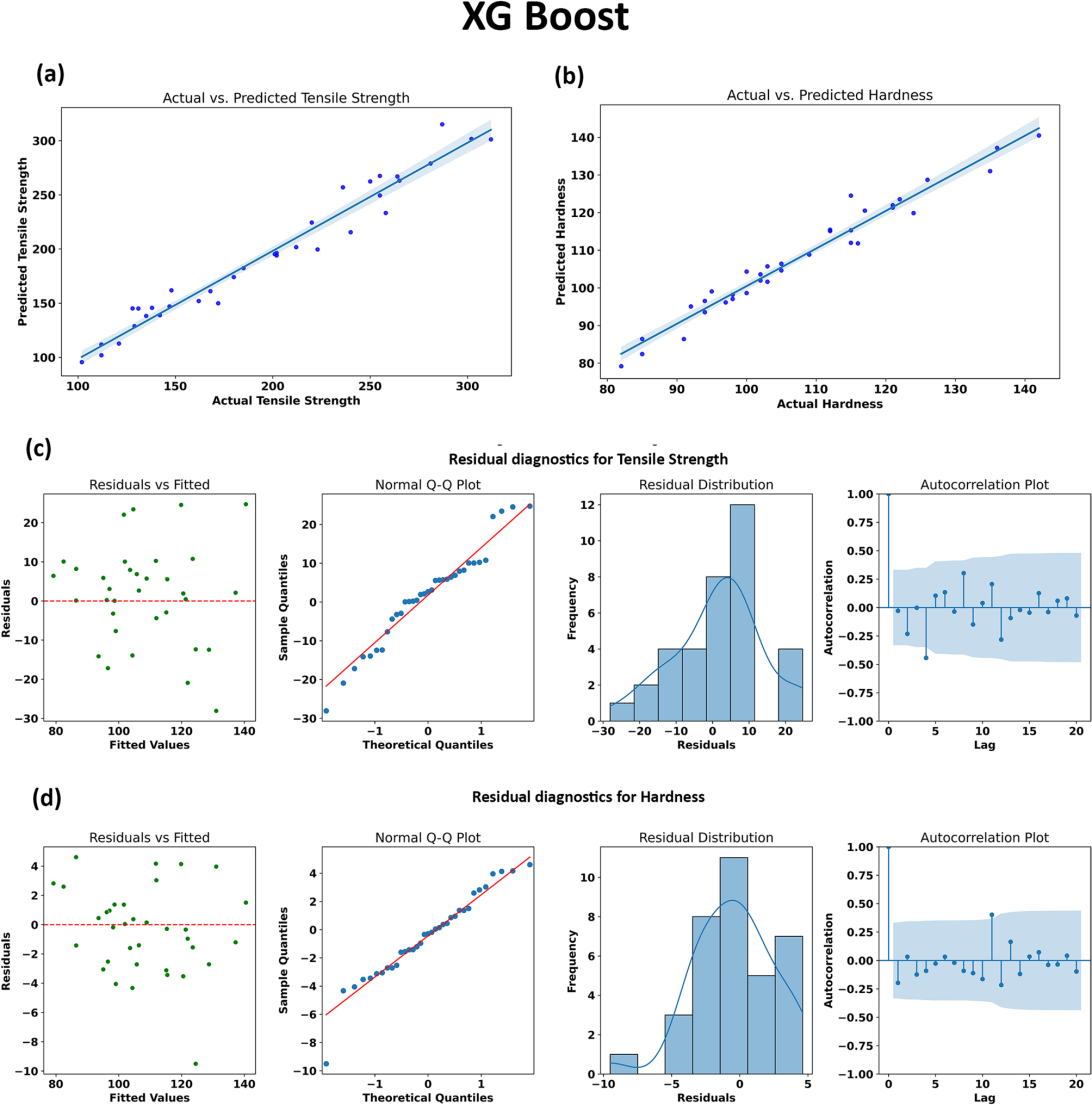
Performance evaluation and residual diagnostics of the XGBoost model for predicting tensile strength and hardness. (a) and (b) show the regression plots comparing actual versus predicted values for tensile strength and hardness, (c) and (d) depict the residual diagnostics for tensile strength and hardness, including residual scatter plots, Q-Q plots, histograms and autocorrelation functions.

In the present study, XGBoost demonstrated outstanding predictive performance for both tensile strength and hardness of AA7075/SiC/ZrC hybrid composites. As visualized in Fig. 14, the regression plots (Fig. 14a and b) show a strong alignment of predicted versus actual values for both target properties, reflected quantitatively by high R^2^ scores (0.9581 for tensile strength and 0.9595 for hardness as previously reported). The close clustering of data points around the diagonal line signifies minimal systematic bias and high predictive fidelity across the entire measurement range.

The residual diagnostics (Fig. 14c and d) further substantiate the reliability of the XGBoost model. The residual scatter plots reveal that prediction errors are randomly distributed and do not exhibit structured patterns, confirming homoscedasticity and suggesting that the model does not systematically under- or over-predict in any region. Q-Q plots demonstrate that residuals closely approximate normality, a key indicator that the model captures the majority of explainable variation without leaving significant structure unexplained. The histograms of residuals are symmetrically centered near zero, reinforcing the model’s unbiased performance. Furthermore, autocorrelation function (ACF) plots indicate the independence of residuals, suggesting that prediction errors are not temporally or sequentially correlated across the dataset.

XGBoost’s robust results with the present dataset highlight its strength in modeling systems characterized by moderate sample size, coupled and nonlinear relationships, and potentially subtle interaction effects among multiple processing and compositional variables. Collectively, these results underscore XGBoost’s status as a leading model for property prediction in materials informatics, especially when interpretability (through features like SHAP values) and accuracy are both required. A more granular analysis of feature importance and model interpretability is discussed in the following sections, further illuminating the value of XGBoost in predictive materials design^69^.

#### Support Vector Regression

Support Vector Regression (SVR) is a kernel-based machine learning technique that aims to find the best-fitting function by maximizing the margin (or “tube”) within which most data points fall, rather than minimizing the error for every sample. By employing the radial basis function (RBF) kernel and tuning the penalty (C) and gamma parameters, SVR is particularly adept at modeling complex, nonlinear relationships in datasets where the underlying process–property connections are not strictly linear. This is especially relevant in advanced composite materials research, where intricate interdependencies between compositional and processing variables are common.

**Fig. 15:**
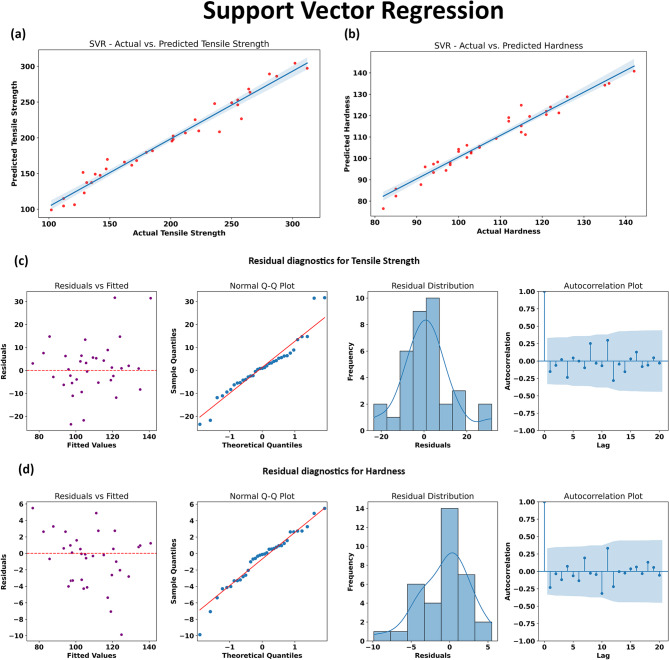
Performance evaluation and residual diagnostics of the Support Vector Regression (SVR) model for predicting tensile strength and hardness. (a) and (b) show the regression plots comparing actual versus predicted values for tensile strength and hardness, (c) and (d) depict the residual diagnostics for tensile strength and hardness, including residual scatter plots, Q-Q plots, histograms and autocorrelation functions.

In the current study, SVR exhibited strong predictive capabilities for both tensile strength and hardness of AA7075/SiC/ZrC hybrid composites. As illustrated in Fig. 15, the regression plots (Fig. 13a and b) demonstrate a close alignment between actual and predicted values across both mechanical properties. Data points are tightly clustered around the diagonal, indicating high model fidelity and low systemic bias over the entire range of observations. This strong agreement is reflected in the quantitative metrics, with SVR achieving some of the highest R² values among all algorithms for both tensile strength and hardness, as previously reported.

The residual diagnostics (Fig. 15c and d) provide further evidence of SVR’s effectiveness. Residual scatter plots for both outputs reveal that errors are randomly distributed around zero, with no visible structure or heteroscedasticity, confirming unbiasedness and robust coverage of the feature space. Q-Q plots show that the residuals closely follow the theoretical quantile line, indicating an approximately normal residual distribution and hence, a lack of unmodeled structure. Histograms centered around zero further support this, suggesting that over- and under-predictions balance out. Autocorrelation plots of residuals display minimal correlation at non-zero lags, indicating temporal and sequential independence—another desirable statistical attribute for reliable predictive modeling.

SVR’s successful performance in this context highlights its strength in handling moderate-sized datasets with nonlinear, intertwined relationships among multiple input parameters. Its ability to capture both global trends and localized variations makes it well-suited to datasets where precise predictions across varying compositional and processing conditions are critical. The consistently strong results from both the regression and diagnostic plots underscore SVR’s value as a robust model for advanced materials data, particularly where model interpretability and prediction accuracy must be balanced.

#### K- Nearest Neighbours

The K Nearest Neighbour (KNN) regression algorithm is a non-parametric, instance-based learning method that estimates the property of a new data point by averaging the known properties of its k closest neighbours in the feature space. In this study, KNN utilized k = 3 with distance-based weighting and Euclidean distance (*p* = 2), ensuring that predictions for tensile strength and hardness were dominated by the most similar prior examples in terms of processing parameters and compositions. This approach is particularly effective for datasets with well-populated, locally smooth regions, as is generally the case here, though KNN’s performance can degrade in the presence of sparse or highly clustered data.

**Fig. 16:**
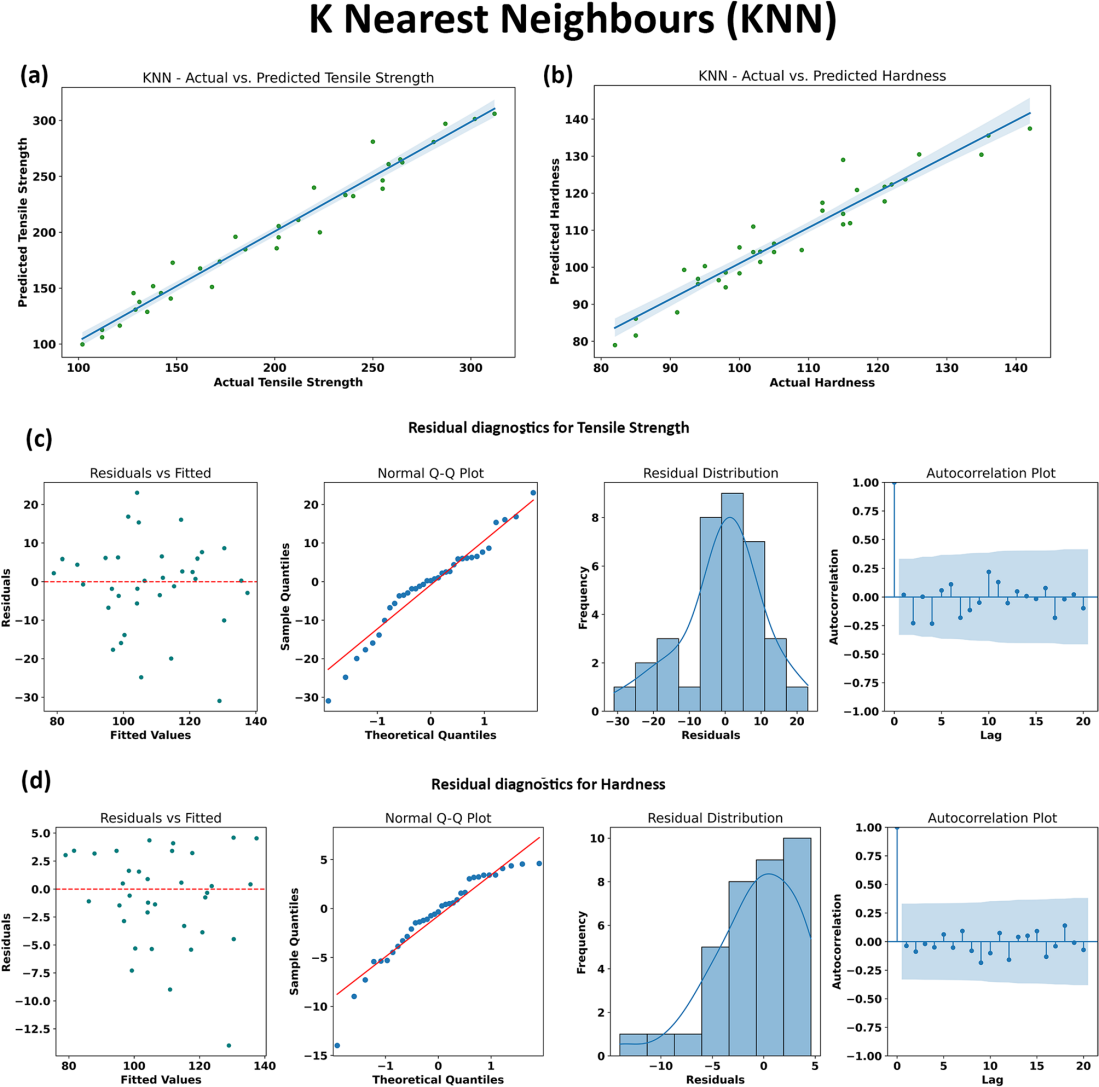
Performance evaluation and residual diagnostics of the K Nearest Neighbour (KNN) model for predicting tensile strength and hardness. (a) and (b) show the regression plots comparing actual versus predicted values for tensile strength and hardness, (c) and (d) depict the residual diagnostics for tensile strength and hardness, including residual scatter plots, Q-Q plots, histograms and autocorrelation functions.

Figure 16 comprehensively illustrates the evaluation of the KNN model for both mechanical properties. The regression plots (Fig. 16a and b) display a strong alignment between the predicted and actual values for both tensile strength and hardness, with points closely tracking the diagonal reference line. This result reflects KNN’s reasonable ability to interpolate within the high-density regions of the processing–property space. The performance metrics reported elsewhere (e.g., R^2^ = 0.9639 for tensile and R^2^ = 0.9163 for hardness) reinforce the strong predictive capacity of KNN, especially for tensile strength, marking it as a competitive method among non-parametric regressors.

Residual diagnostics further support these findings. The scatter plots of residuals versus fitted values indicate a random, patternless distribution around zero for both outputs, suggesting minimal systematic bias and good model calibration across the range of predicted values. The normal Q-Q plots show that most residuals fall along the theoretical quantile line, with only minor deviations in the tails, indicating an approximately normal error distribution and the absence of strong outliers. The residual histograms are symmetric and centralized, again confirming unbiased predictions. Lastly, autocorrelation plots reveal no significant lag-dependent residual structure, implying statistical independence in prediction errors.

Collectively, these results demonstrate that the KNN model is an effective choice for property prediction in this materials context, particularly where the dataset is sufficiently dense and locally homogeneous. KNN’s strengths lie in its intuitive operation and lack of assumptions about functional form; however, its predictive accuracy may be limited in under-sampled regions or where more global, highly nonlinear relationships exist. Nonetheless, for the present AA7075/SiC/ZrC hybrid composite dataset, KNN delivers accurate, resilient predictions with well-behaved residuals, as visualized in Fig. 16, and provides a useful benchmark against more complex machine learning algorithms discussed elsewhere in this work.

#### Artificial Neural Networks (ANN)

Artificial Neural Networks (ANNs) are powerful data-driven models inspired by the human brain’s structure, adept at capturing complex, nonlinear relationships in multidimensional datasets. In this study, the ANN architecture employed consisted of a single hidden layer with 100 neurons and the ‘tanh’ activation function, optimized for both predictive capacity and interpretability across the hybrid composite dataset. ANNs excel at modeling intricate, high-dimensional mappings—an essential quality for materials systems where property responses depend on subtle, interacting effects among several compositional and processing variables.

**Fig. 17:**
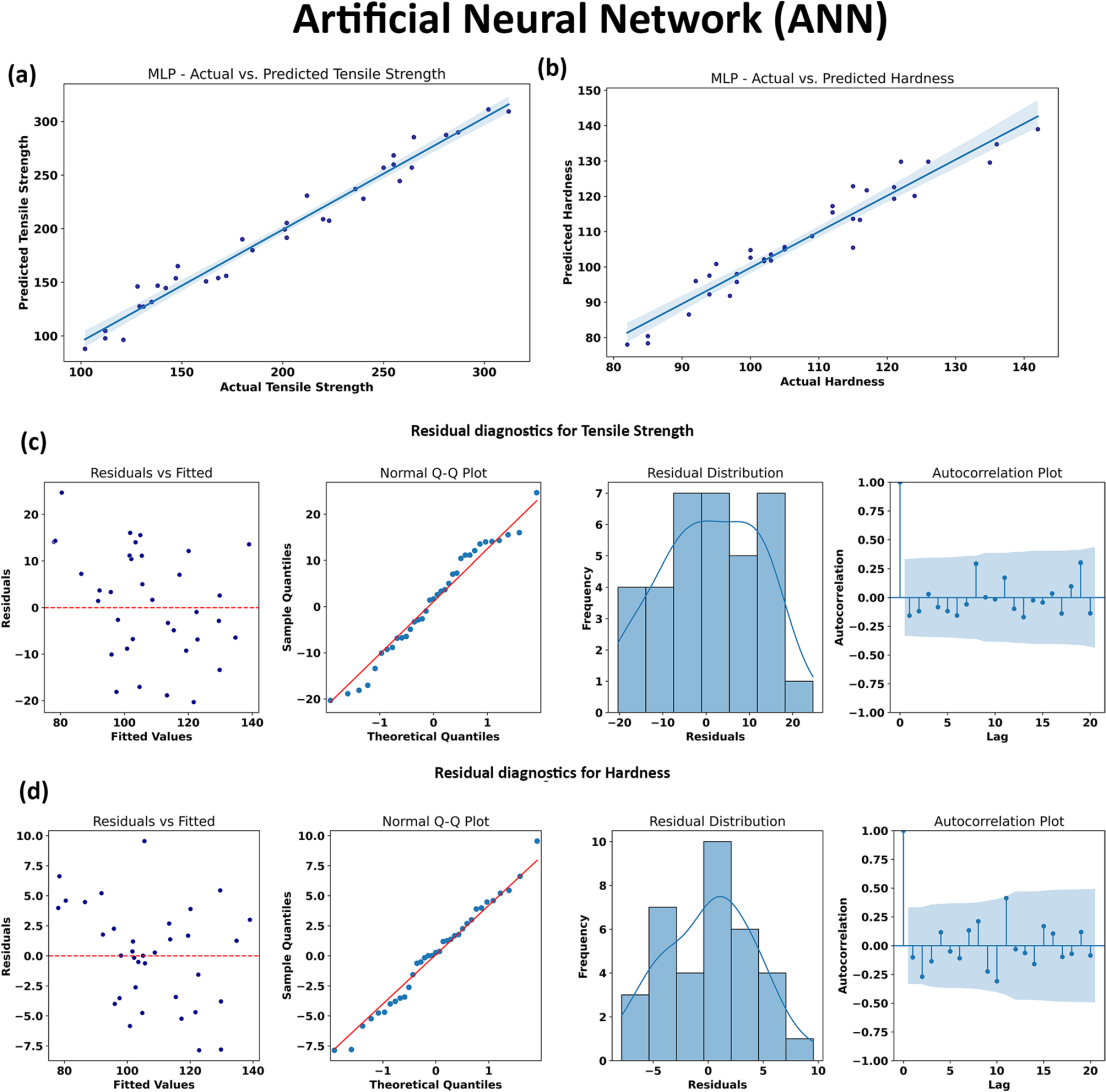
Performance evaluation and residual diagnostics of the Artificial Neural Network (ANN) model for predicting tensile strength and hardness. (a) and (b) show the regression plots comparing actual versus predicted values for tensile strength and hardness, (c) and (d) depict the residual diagnostics for tensile strength and hardness, including residual scatter plots, Q-Q plots, histograms and autocorrelation functions.

As illustrated in Fig. 17, the ANN model delivers excellent predictive accuracy for both tensile strength and hardness. The regression plots (Fig. 17a and b) show an almost ideal alignment of predicted versus actual values, with data points densely distributed along the diagonal reference line for both output properties. This result is consistent with the high R^2^ value achieved in prior evaluations (e.g., R^2^ = 0.9748 for tensile strength), indicating that the ANN effectively learns both the linear and nonlinear components of the materials property landscape.

The residual diagnostics in Fig. 17c and d further confirm the ANN’s robust performance. Residual scatter plots reveal a random dispersion of errors around zero with no clear patterns or trends, demonstrating that the model is not biased towards any particular region of the property space and maintains reliable accuracy across the range of predicted values. Normal Q-Q plots for both properties closely follow the theoretical line, indicating that residuals are approximately normally distributed and that the model has captured most of the systematic variation in the data. Symmetrical and narrow histograms centered around zero further support the absence of significant bias and the general randomness of the errors. Finally, autocorrelation function plots confirm that residuals are not serially correlated, suggesting statistical independence of errors and reinforcing the model’s ability to faithfully represent the underlying data.

Overall, the ANN model demonstrates outstanding predictive power for the AA7075/SiC/ZrC hybrid composite dataset, accurately reproducing both tensile strength and hardness across a diverse set of experimental conditions. Its ability to handle complex, high-dimensional relationships makes it especially suitable for advanced materials design, while the residual diagnostics provide compelling evidence of model validity and reliability. A broader discussion of the comparative strengths, limitations, and interpretability of ANN and other machine learning algorithms is presented in the following sections.

### Comparative analysis of machine learning models

**Fig. 18:**
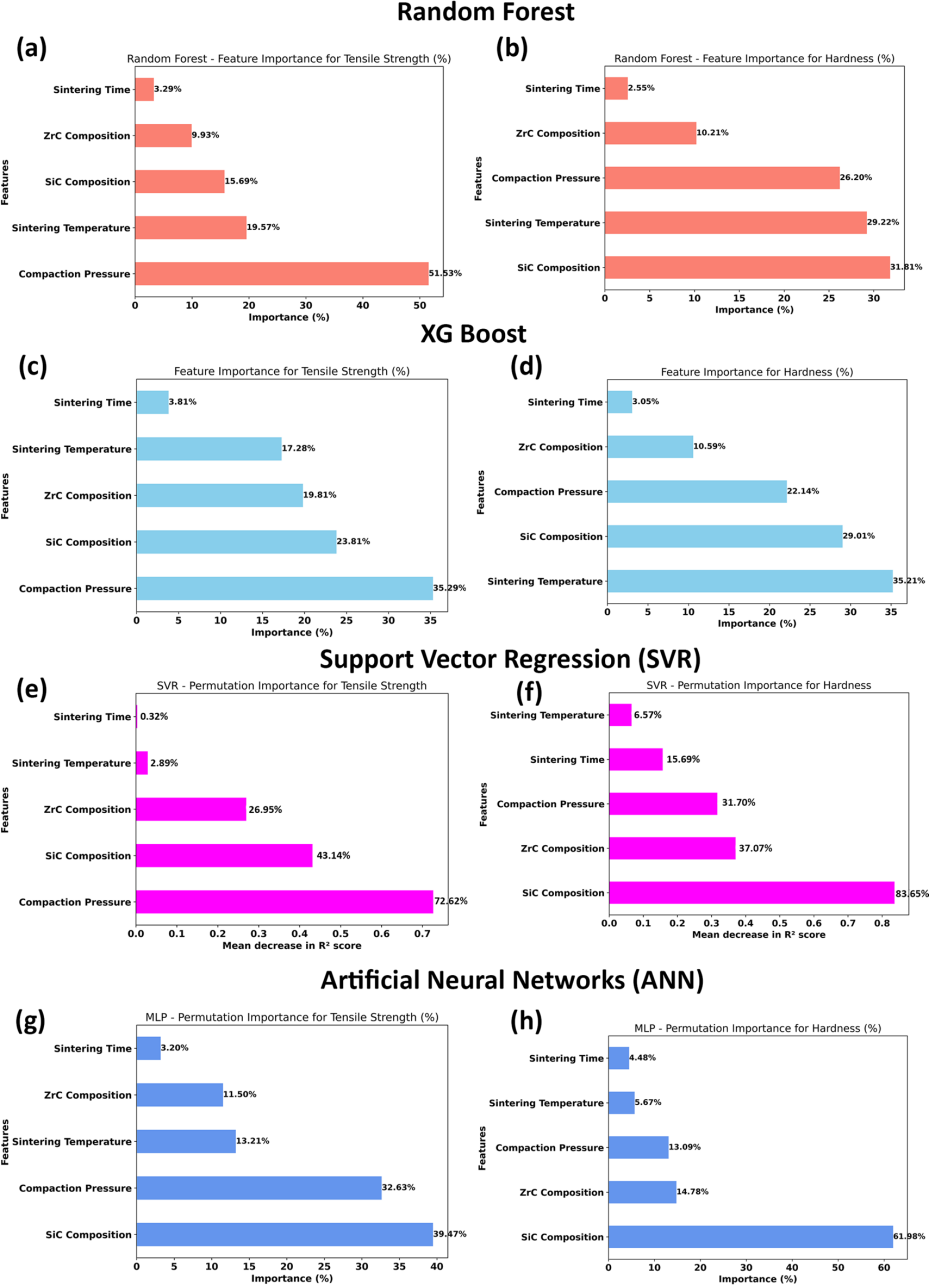
Comparison of feature importance across different machine learning models for predicting tensile strength and hardness.

Figure 18 presents a side-by-side comparison of feature importance rankings as determined by Random Forest, XGBoost, Support Vector Regression (SVR), and Artificial Neural Network (ANN) models for both tensile strength and hardness. This comparative visualization offers critical insight into each algorithm’s internal decision processes and elucidates how well the models’ focus aligns with established metallurgical understanding.

For tensile strength prediction, Random Forest and SVR models both assign predominant importance to compaction pressure (over 50% for Random Forest, over 70% for SVR), strongly suggesting that densification during powder compaction is the overriding factor governing load-bearing capacity in these microwave-sintered composites. XGBoost and ANN also recognize the substantial influence of compaction pressure, but distribute importance more evenly among SiC composition, sintering temperature, and ZrC composition—reflecting their enhanced capacity to capture complex, multi-factor interactions. Notably, SiC composition consistently emerges as a major contributor in all models except SVR, underscoring its mechanical role: SiC serves as the primary reinforcement, directly improving tensile strength by enabling load transfer from the ductile matrix and providing Orowan strengthening and grain refinement. Sintering temperature also features prominently (especially in XGBoost), reflecting its control over microstructural cohesion and residual porosity.

For hardness prediction, a shift in importance is observed. Both Random Forest and ANN models attribute their highest weight to SiC composition (over 30% for RF, over 60% for ANN), aligning with the mechanical reality that surface hardness in aluminium matrix composites is most strongly governed by the presence, distribution, and effective bonding of hard ceramic phases. The feature importance for sintering temperature and compaction pressure remains significant in most models, reinforcing the role of process-driven densification and phase evolution in maximizing indentation resistance. Interestingly, SVR identifies SiC composition as overwhelmingly dominant (over 80%), and XGBoost shows a relatively balanced importance between SiC, sintering temperature, and compaction pressure, indicative of its ability to parse non-linear and interaction effects.

Interpreting these trends alongside the model performance metrics provides further insight. Models like ANN and XGBoost, which effectively distribute importance across multiple relevant features and capture their interactions, achieved the best prediction scores—ANN for tensile strength (R² = 0.9748, lowest MAE/RMSE) and XGBoost for hardness (R^2^ = 0.9595, lowest MAE/RMSE). This superior accuracy stems from their ability to mimic the true, multi-causal nature of mechanical property development: optimal mechanical performance in hybrid MMCs is not due to a single factor, but results from synergy between reinforcement content, compaction, and sintering. In contrast, models such as SVR, which concentrated their importance too narrowly (especially for hardness), may overlook the impact of process variables on microstructural uniformity—potentially explaining their marginally lower hardness prediction accuracy despite excellent results for tensile strength, where the major control (compaction pressure) is correctly identified.

A key strength of the present study lies in the consistency between machine learning predictions and experimental microstructural observations. The feature importance analysis identified compaction pressure as the dominant factor influencing tensile strength, which aligns with microstructural evidence showing improved densification and reduced porosity at higher compaction pressures. Similarly, the strong influence of SiC composition on microhardness predicted by the models is supported by SEM observations, where uniformly distributed SiC particles contribute to enhanced hardness through load transfer and Orowan strengthening mechanisms.

Furthermore, the influence of sintering temperature captured by the machine learning models corresponds closely with observed changes in microstructure, including improved interfacial bonding at optimal temperatures and grain coarsening at higher temperatures. The reduced effectiveness of ZrC at higher contents, as indicated by model predictions, is also consistent with microstructural observations of particle agglomeration and associated defects. These correlations demonstrate that the machine learning models are not only statistically accurate but also physically meaningful, as they successfully capture the underlying microstructure–property relationships governing the behavior of AA7075/SiC/ZrC hybrid composites.

In summary, the feature importance rankings confirm that SiC composition is the primary driver of hardness, while compaction pressure—closely followed by SiC content and sintering parameters—dominates the development of tensile strength. Models that reflect this balance in their learned importance, particularly ANN and XGBoost, provide the most accurate and physically relevant predictions. This alignment of machine learning interpretation with materials science fundamentals underscores the reliability of the models and builds confidence in their use for guiding future composite design and process optimization.

## Conclusions

This study systematically demonstrated the efficacy of machine learning (ML) algorithms for the predictive modeling of microhardness and tensile strength in microwave-sintered AA7075/SiC/ZrC hybrid composites, using a rigorously curated dataset spanning 172 experimental conditions and encompassing key process variables such as SiC and ZrC compositions, compaction pressure, sintering temperature, and sintering time. Extensive data visualization—ranging from box and violin plots to advanced multidimensional projections—facilitated an in-depth understanding of data distributions, variable interactions, and the influence of process design on measured properties.

Among the evaluated models, Artificial Neural Networks (ANN) and XGBoost exhibited outstanding predictive performance—ANN achieving the highest accuracy for tensile strength, while XGBoost provided the best results for microhardness prediction. Random Forest, KNN, and SVR also demonstrated strong predictive capability, with their relative accuracy reflected in their ability to capture both simple and complex non-linear relationships within the dataset. Residual diagnostics confirmed that the best-performing models were well-calibrated, with minimal bias, random error distribution, and no significant signs of overfitting.

Model interpretability through feature importance analysis revealed that microhardness is primarily governed by SiC content and sintering parameters, while tensile strength is strongly influenced by compaction pressure, SiC composition, and optimal sintering conditions. The consistency between the models’ feature rankings and established metallurgical mechanisms—such as load transfer, Orowan strengthening, grain refinement, and the adverse effects of overloading reinforcement—confirms the physical reliability and scientific value of the predictions.

Microstructural investigations corroborated these data-driven insights: uniform dispersion of reinforcements, clean matrix–particle interfaces, and controlled grain size collectively led to property enhancements, while agglomeration (particularly above 3–4% ZrC) and porosity at high reinforcement contents diminished mechanical response. The comprehensive integration of data-driven modeling and materials characterization provides a blueprint for optimizing the fabrication and design of new hybrid metal matrix composites with tailored properties.

Overall, this work highlights the potential for advanced ML models—especially tree-based ensembles and deep neural networks—to serve as reliable, interpretable, and efficient tools in accelerated materials design. By substantially reducing experimental workload and guiding the selection of optimal processing routes, these approaches pave the way for rapid and cost-effective development of next-generation lightweight, high-performance composite materials.

While the present study demonstrates the effectiveness of ensemble-based machine learning models in predicting mechanical properties, future work can focus on the development of hybrid physics-informed machine learning frameworks. Such approaches would enable the incorporation of underlying metallurgical principles—such as strengthening mechanisms, diffusion behavior, and densification kinetics—directly into the learning process. This integration has the potential to further enhance predictive accuracy, interpretability, and reliability for complex materials systems.

## Data Availability

The experimental dataset generated and analyzed during this study is available from the corresponding author upon reasonable request. The present work forms the basis for further development of advanced physics-informed machine learning models. Upon completion of these extended studies, the dataset and corresponding model implementation will be curated and made publicly available through appropriate repositories to ensure transparency and reproducibility.
